# Automatic extraction of SmPC document for IDMP data model construction using foundation LLM and RAG: a preliminary experiment for pharmaceutical regulatory affairs

**DOI:** 10.3389/fmed.2025.1598979

**Published:** 2025-08-13

**Authors:** Hocine Kadi, Alaa Abdellatif, Daniel Isaac Kemajou Njamen, Florian Pereme

**Affiliations:** ^1^Product Life Group, Courbevoie, France; ^2^Pharma IT, Product Life Group Company, Copenhagen, Denmark

**Keywords:** IDMP, SmPC extraction, pharmaceutical regulatory affairs, LLM, RAG, NLP, data standardization, regulatory compliance

## Abstract

**Introduction:**

The pharmaceutical industry is undergoing a significant shift from traditional paper-based processes to data-driven approaches. This transition necessitates the adoption of structured data-exchange standards, such as the IDentification of Medicinal Products (IDMP), to improve harmonization, transparency, and interoperability across global regulatory landscapes. However, transforming unstructured data, such as Summary of Product Characteristics (SmPC) documents, into structured IDMP models presents considerable challenges in data extraction and standardization.

**Methods:**

We investigated the application of foundation Large Language Models (LLMs), namely Claude 3.5 Sonnet and Gemini 1.5 Flash, combined with Retrieval-Augmented Generation (RAG) techniques. We utilized various embedding models (generalist, specialized, and hybrid) and rule-based retrieval approaches. To improve the precision of the information extracted from the medicinal product from the SmPC documents, we evaluated multiple prompting strategies.

**Results:**

Our investigation showed that Claude 3.5 Sonnet significantly surpassed Gemini 1.5 Flash in performance. Additionally, RAG-type approaches with semantic research using embedding models were superior to rule-based methods overall. The choice of embedding models was essential depending on the type of information being extracted. Prompts that incorporated context, action, and examples were more effective than those based solely on role and steps. The approach achieved a BERT F1 score of up to 0.98 for the medicinal product section.

**Conclusion:**

Our findings demonstrate that the proposed LLM-RAG approach enables accurate and scalable extraction of structured data from SmPCs. This supports the digital transformation of regulatory processes by promoting standardization, interoperability, and harmonization. If implemented effectively, the method could help pharmaceutical companies improve regulatory compliance, streamline submissions, and improve data consistency.

## 1 Introduction

The pharmaceutical industry is undergoing a significant digital transformation, transitioning from traditional paper-based processes to advanced digital methodologies. A key aspect of this evolution is the implementation of the IDentification of Medicinal Products (IDMP) standards introduced by the International Organization for Standardization (ISO) (1). These standards aim to digitize all medicinal product information into a structured and harmonized data model, enhancing efficiency, interoperability, and data quality across the industry.

Historically, medicinal product data have been stored in unstructured, static documents such as PDFs, notably Summary of Product Characteristics (SmPCs). SmPCs provide comprehensive details on medicinal products, including composition, clinical data, pharmacological properties, and therapeutic indications (2). Although they ensure reliable information for healthcare professionals, the nature of free natural language text of SmPCs limits their adaptability, accessibility, and integration into digital systems. This rigidity poses challenges in today's fast-paced global healthcare landscape, where timely and accurate information exchange is crucial.

The application of artificial intelligence (AI) in healthcare and pharmaceuticals has expanded rapidly in recent years (3, 4), transforming the way data are processed and analyzed. This expansion has led to better decision-making and patient care. AI technologies such as machine learning (ML) and large language models (LLMs), like GPT, Claude, and Gemini, have proven valuable in enhancing diagnostic accuracy (5), optimizing treatment protocols, and expediting drug discovery processes. However, the use of LLMs presents certain risks. Specifically, there is a possibility that they may generate “hallucinations,” producing inaccurate or misleading information (6, 7). Hallucinations can have severe implications in critical fields such as medicine and pharmaceuticals.

To mitigate these risks, approaches such as prompt engineering and Retrieval-Augmented Generation (RAG) have been developed. Prompt engineering involves crafting specific inputs to optimize LLM performance, guiding the model to produce more accurate and reliable outputs in tasks such as diagnosing conditions from medical images or generating medical reports (8). RAG is a technique that combines real-time data retrieval with LLM outputs to enhance accuracy (9). Incorporating RAG into these tasks provides an additional layer of accuracy by cross-referencing real-world data, making LLM outputs more reliable for clinical and pharmaceutical applications. This technique is crucial in healthcare settings where the cost of inaccuracies can be extremely high. Using both prompt engineering and RAG, LLMs can improve efficiency and reliability across a range of tasks, from diagnosing diseases to supporting personalized care and regulatory compliance.

This study aims to explore the capability to rebuild the IDMP data model from regulatory affairs free text documents using LLM applications. We especially aim to:

1. **Build a minimal IDMP structure**: represent essential data fields to accurately identify medicinal products, ensuring alignment with the EU IDMP implementation guide (IG) (10). The concept of “minimal IDMP” refers to data fields that cannot be consolidated or retrieved afterward from other structured data sources. It is basically the unique key combination of information that will allow us to cross-retrieve the rest of the full IDMP model content.
2. **Leverage LLM and RAG methods**: employ models such as Claude 3.5 and Gemini 1.5 to interpret complex medical texts within SmPCs, combining them with RAG techniques to enhance data extraction accuracy.
3. **Evaluate effectiveness**: assess the effectiveness of RAG LLM strategies in processing SmPC data, focusing on their ability to handle unstructured information, mitigate issues like hallucinations, and maintain high precision. We compared rule-based retrieval and semantic search with embedding techniques to determine the best approach for extracting relevant information from SmPC documents.

Given these challenges, this study addresses four research questions:

- **RQ1**: Can an LLM-RAG system efficiently extract IDMP-relevant data from unstructured SmPC files?
- **RQ2**: What is the optimal combination of LLM with retrieval techniques, including rule-based retrieval and semantic search with embedding techniques, in a RAG system for extracting domain-specific information (IDMP) from unstructured SmPCs?
- **RQ3**: Which embedding model type, generalist or specialized, is more effective in a RAG system for extracting IDMP data from unstructured SmPC files?
- **RQ4**: What are the most effective prompts for guiding an LLM to extract IDMP-relevant data from unstructured SmPC files?

The rest of this paper is organized as follows. In Section 2, we review related work on LLMs, prompt engineering, and RAG specifically within the healthcare and pharmaceutical domains. Section 3 outlines the material and methods used in this study, including data collection, LLM and prompt selection, the implementation of the LLM-RAG system architecture, and the evaluation metrics. Section 4 presents the experimental results, focusing on the performance of the LLM-RAG system in extracting IDMP-relevant data from SmPCs. Section 5 provides an in-depth analysis of the findings, addressing the research questions and comparing our results with existing approaches in the healthcare and pharmaceutical contexts. Finally, Section 6 concludes the paper by summarizing the key contributions and suggesting directions for future research in these critical domains.

## 2 Related works

### 2.1 Large language models in healthcare and pharmaceutical

Generative AI (Gen AI) is transforming healthcare by optimizing clinical workflows and enhancing patient care through various impactful applications (11). These AI-driven models empower healthcare professionals to extract valuable insights from extensive datasets, such as medical literature and patient records, by leveraging advanced prompting techniques (8). Applications like question-answering systems, text summarization, and machine translation streamline the processing of information, enabling more efficient data utilization.

In pharmacovigilance, Gen AI automates and refines processes like aggregate reporting, signal detection, and safety surveillance by handling large, complex datasets from sources like electronic health records and social media, addressing traditional challenges of labor-intensive data handling and delays in signal detection (12). In drug development and regulatory science, AI enhances pharmacovigilance, adverse event reporting, and drug design, analyzing diverse data with remarkable speed and precision. Developing transparent guidelines around data quality, model interpretability, and ethical use is essential for regulatory agencies to fully harness AI's potential. Coordinated international standards will support a balanced framework that advances drug safety, innovation, and patient outcomes across all stages of drug development (13).

Studies have shown the effectiveness of LLMs in various healthcare applications. Gunes and Cesur (14) found that Claude 3 Opus outperformed other LLMs and general radiologists in diagnostic accuracy for thoracic radiology cases. Rewthamrongsris et al. (15) evaluated LLMs in answering questions on antibiotic prophylaxis for infective endocarditis during dental procedures, finding that pre-prompts generally enhanced accuracy. Tang et al. (16) investigated LLMs in extracting key medical information from research papers, with GPT-4.0 outperforming GPT-3.5. Silberg et al. (17) presented UniTox, a dataset for drug-induced toxicities, achieving high accuracy and clinician validation.

The adoption of LLMs and Gen AI in healthcare and pharmaceutical fields is becoming indispensable. However, challenges remain, such as refining prompting techniques, enhancing model fine-tuning, and implementing advanced approaches like RAG.

### 2.2 Prompt engineering in healthcare and pharmaceutical

Automated prompts are generated using algorithms, enhancing efficiency and adaptability, and are categorized into discrete and continuous prompts. Prompt engineering is crucial for optimizing LLM interactions with unstructured data. It involves crafting queries to enhance the LLM's ability to retrieve and process information efficiently. Various prompt types include cloze prompts, prefix prompts, manual prompts, automated prompts, zero-shot prompting, and few-shot prompting, each with unique benefits and limitations depending on the task's complexity (8).

Zero-shot prompting uses a well-designed prompt without examples, relying on the model's pre-trained knowledge. Few-shot prompting includes a few examples to guide the model's responses, improving accuracy for specific outputs. Tang et al. (16) evaluated GPT-3.5 and GPT-4.0 in extracting medical information, revealing that prompt engineering strategies can positively influence model performance but vary depending on the task and GPT version. Combining multiple prompt strategies did not always yield better results; simpler prompts sometimes outperformed complex combinations. GPT-3.5 favored the persona strategy, while GPT-4.0 showed better results with few-shot prompting.

Nori et al. (18) explored whether generalist foundation models like GPT-4 can outperform specialist models without intensive domain-specific tuning. Their case study in medicine demonstrated that through innovative prompt engineering, generalist models could not only match but surpass the performance of specialized models such as Med-PaLM 2 on various medical question-answering benchmarks. By introducing the Medprompt strategy a composition of dynamic few-shot selection, self-generated chain-of-thought reasoning, and choice shuffle ensembling they achieved state-of-the-art results across nine benchmark datasets in the MultiMedQA suite. This study highlights that prompt engineering can unlock deeper specialist capabilities in generalist models, reducing the need for extensive fine-tuning and expert-crafted prompts.

Zhou et al. (19) introduced the LEAP Framework for clinical relation extraction, integrating adaptive prompts to enhance LLM performance in extracting clinical relationships from medical texts. This framework significantly improves relation extraction tasks across multiple datasets and models, achieving superior *F*1 scores compared to traditional methods. This work aligns with findings from Tang et al. (16) and Nori et al. (18), highlighting the importance of sophisticated prompt engineering in optimizing LLM performance for complex tasks. Kartchner et al. (20) explored zero-shot information extraction for clinical meta-analyses of RCTs, demonstrating that models like ChatGPT can effectively extract clinical data from RCT abstracts without prior task-specific training. By using tailored prompt-based strategies, researchers achieved high accuracy in identifying key clinical parameters. The study also noted strengths and limitations of zero-shot approaches, including the ability to recognize missing information and the prevalence of errors like verbosity and hallucinations.

These studies collectively underscore the critical role of prompt engineering in maximizing LLM potential for medical data extraction and other specialized applications. Both the selection of appropriate prompt strategies and the model's inherent capabilities are crucial for optimal performance.

### 2.3 RAG in medical and pharmaceutical applications

RAG combines LLM capabilities with retrieval mechanisms to enhance accuracy, relevance, and reliability in medical and pharmaceutical applications. This is crucial in healthcare and regulatory compliance, where incorrect information can have serious consequences. Wang et al. (21) developed BioRAG, which combines LLMs with domain-specific models like PubMedBERT to improve the accuracy of medical information retrieval. It dynamically incorporates external biomedical knowledge, providing precise and contextually relevant outputs, particularly useful in pharmacovigilance and regulatory submissions. BioRAG minimizes hallucinations and improves real-time decision-making, handling vast unstructured biomedical data such as clinical trial results and adverse drug event reports.

Jiang et al. (22) proposed TC-RAG, a Turing-complete system that improves retrieval control by using memory stacks, thereby mitigating the accumulation of outdated or irrelevant information. It enhances data extraction accuracy and control, especially in large, evolving healthcare datasets like clinical trials and patient records. MedGraphRAG (23) is a graph-based extension of RAG that improves the safety and reliability of LLM-generated medical responses by linking medical entities to a hierarchical graph structure grounded in credible sources. Kim and Min (24) QA-RAG presented a dual-track retrieval system combining queries with answers generated by a fine-tuned LLM, ensuring accurate and contextually relevant document retrieval. Choi et al. (25) demonstrated MALADE, showcasing the power of multi-agent systems powered by RAG for ADE extraction. By orchestrating multiple LLM agents, MALADE effectively extracts and analyzes ADE data from large, unstructured sources such as FDA drug labels and electronic health records, providing both qualitative and quantitative insights for drug safety and adverse event monitoring. TC-RAG (22) improves retrieval control using memory stacks, enhancing data extraction accuracy in healthcare datasets.

### 2.4 Generalist vs. specialized embedding models in RAG systems

Embedding techniques are crucial for enhancing systems in healthcare, pharmaceuticals, and regulatory compliance, where accuracy and relevance are vital. Recent studies highlight the importance of embeddings in optimizing semantic search and retrieval, showing that tailored strategies significantly improve performance. Amugongo et al. (26) reviewed RAG in healthcare and emphasized the use of dense embeddings to enhance retrieval accuracy and reduce hallucinations. Models like OpenAI's ADA-002 enhance the relevance of clinical information, supporting applications such as clinical question-answering and diagnostics. The review categorizes RAG techniques into “Naive,” “Advanced” and “Modular,” each progressively using embeddings to provide contextually relevant responses in healthcare.

Chen et al. (27) introduced PharmaGPT, which leverages custom embeddings to improve retrieval for bio-pharmaceutical and chemical tasks, excelling at bio-pharma benchmarks like NAPLEX. These embeddings enhance retrieval precision for specialized queries, highlighting their utility in RAG applications within specialized fields. QA-RAG (24) uses dense embeddings in regulatory compliance for the pharmaceutical industry, combining FAISS indexing with dense embeddings to accurately retrieve complex regulatory documents, such as FDA and ICH guidelines. This approach reduces compliance risks and supports highly accurate regulatory decisions by grounding responses in reliable and current information. Wu et al. (28) proposed AskFDALabel, a framework built for the FDA's regulatory tasks, integrates a semantic search module based on sentence embeddings and a Q& A generation module for drug labeling. Using Sentence Transformer embeddings optimized with metadata for entity recognition, AskFDALabel improves retrieval accuracy and transparency. This framework improves decision making in drug review processes by providing secure, computationally efficient, RAG-based support tailored to regulatory requirements.

Excoffier et al. (29) compared generalist and specialized embedding models in clinical semantic search tasks, such as matching rephrased ICD-10-CM code descriptions. Evaluating 19 models on 1, 000 reformulated descriptions, they found generalist models like jina-embeddings-v2-base-en and e5-small-v2 outperformed specialized models like ClinicalBERT in short-context tasks. The top generalist model achieved an 84.0% exact matching rate, compared to 64.4% for the best specialized model. Generalist models' superior performance was due to training on larger, more diverse datasets, enhancing robustness against text variations. However, specialized embeddings may still excel in tasks requiring deep contextual understanding of complex clinical narratives. This research highlights the importance of selecting embedding models based on the retrieval task's characteristics, such as input text length and complexity.

According to Setty et al. (30), embedding optimization through refined chunking and metadata annotation significantly enhances retrieval accuracy for finance-specific queries in RAG systems. Financial RAG systems often face retrieval challenges due to the specific jargon and context required, which dense embeddings help address by enhancing the relevance and context in retrieved information. These adjustments increase the system's ability to retrieve high-quality, targeted responses, supporting decision-making in finance.

Finardi et al. (31) investigates RAG in Brazilian Portuguese, evaluating sparse and dense retrieval methods, as well as chunking strategies, to refine retrieval in multilingual settings. This study illustrates how embedding-driven retrieval influences output quality, demonstrating the effectiveness of dense embeddings across language variations. By experimenting with naive and advanced RAG techniques, this study underscores the adaptability of dense embeddings for RAG in non-English applications, contributing to language-specific model optimization.

Caspari et al. (32) explored best practices for embedding use in RAG, showing that techniques such as query transformations, hierarchical chunking, and multimodal retrievalimprove both processing efficiency and response quality. By fine-tuning embeddings within RAG workflows, this research shows how balancing retrieval accuracy with processing efficiency can adapt RAG to a variety of applications with different input types, including visual and text-based content.

Across the reviewed studies, several strategies have been explored to enhance the application of AI in healthcare and pharmaceutical domains. Among these, three particularly relevant and complementary approaches stand out: direct use of LLM, prompt engineering, and RAG. Direct LLM applications facilitate the extraction of information from unstructured data and demonstrate strong diagnostic capabilities [for example, Claude 3 Opus in thoracic imaging (14)], although they may require fine-tuning and can yield variable outputs. Prompt engineering, through approaches such as zero-shot, few-shot, and chain-of-thought prompting, has been shown to improve model performance without retraining (18). However, increasing prompt complexity can sometimes reduce consistency and interpretability. RAG methods combine LLMs with external retrieval systems to ground model outputs in verifiable sources, which helps reduce hallucinations and improve domain-specific accuracy [as seen in BioRAG for pharmacovigilance (21)]. These systems, however, introduce added complexity and depend heavily on the quality of the retrieval pipeline. Embedding strategies also play a critical role. Generalist embeddings tend to perform well in short-context semantic tasks [as shown by Excoffier et al. (29), where models like jina-embeddings-v2 and e5-small-v2 outperformed specialized models such as ClinicalBERT in matching rephrased ICD-10-CM code descriptions], while domain-specific embeddings may still be preferable for tasks requiring deeper contextual understanding.

These insights highlight the importance of integrating advanced prompting techniques, effective retrieval mechanisms, and well-matched embedding models to sup.

## 3 Materials and methods

### 3.1 Building an extraction model

To effectively extract data from SmPC documents, we developed an extraction model based on the notion of the minimal IDMP, defined as a fundamental set of data fields essential for accurately identifying medicinal products. Derived from Chapter 2 of the EU IDMP Implementation Guide [see (10)], these fields establish the baseline for data extraction from SmPC. The root keys of the minimal IDMP are directly derived from the section names listed in the guide, namely:

- Section 1. Medical product (foundational fields are inherently precise and therefore can be extracted from the SmPC without complexity);
- Section 2. Marketing authorization information (regulatory fields capture all the medicinal product information required to comply with the regulatory requirements for market entry and maintenance and ensure compliance across jurisdictions, ensuring extraction aligns with EU-level and national-level requirements);
- Section 3. Therapeutic indications (fields cover the known therapeutic indications for which the medicinal product is authorized. This defines the scope of clinical use and compliance with approved indications as stated in regulatory documents like the SmPC);
- Section 4. The packaged medicinal product (fields reflect the hierarchy of packaging information from the overall package description to the item components and materials used. This nested structure captures the full configuration of the packaged product, ensuring traceability of individual packaging units);
- Section 5. The ingredients (this section details the chemical composition of the medicinal product, including active ingredients and excipients. The complexity of ingredient information extraction arises from the varying ways that pharmaceutical strengths are presented whether by concentration, unit dosage, or overall presentation);
- Section 6. The pharmaceutical product (this section ensures that all product configurations are well documented to align with regulatory and clinical requirements. For instance, the dose form can affect the efficacy of the medicinal product).

This information ensure adherence to the required data elements for the electronic submission of medicinal product information. Keys and nested keys within these root categories are meticulously mapped to the necessary data elements, comprehensively capturing each aspect of a product's identity and characteristics. Table 1 presents the fields constituting the minimal IDMP, along with their corresponding levels of nesting complexity. For more detailed explanation on the contain of the fields see Annex 1. The notion of “Level” in this context reflects the degree of abstraction required by a language model to extract the relevant information.

- **Level 1** includes fields that are *explicitly stated* and can be *directly extracted* from the source text without contextual interpretation. For example, the *Product Name*, such as “*Pixuvri 29 mg powder for concentrate for solution for infusion,”* appears in **Section 1** (Name of the Medicinal Product), and the *ATC Code*, such as “*ATC Code: L01DB11,”* is found in **Section 5.1** (Pharmacodynamic Properties).
- **Level 2** covers fields that contain *multiple sub-elements* requiring basic structuring. A representative example is the *Marketing Authorization Holder* in **Section 7**, where the model must segment components such as the company name (“Les Laboratoires Servier”), address (“50, rue Carnot”), postal code (“92284 Suresnes cedex”), and country (“France”) from a free-text block.
- **Level 3** involves *semantic reasoning across multiple sections* and requires the model to differentiate between structurally similar but semantically distinct fields. In particular, the model must distinguish between *Composition Active* and *Composition Excipient*, and further differentiate within the active composition between *Active Substance Salt* and *Active Substance Base*. For each, it must accurately extract both the *Value* and the corresponding *Dosage*. These values often appear in formulations spread across **Sections 3** (Pharmaceutical Form) and **6.1** (List of Excipients). For example, the model should align the text “*Each tablet contains 5 mg of aripiprazole”* with the active substance and link “*Excipient with known effect: 28 mg of maltose per tablet”* to the correct formulation and role. This level of complexity requires entity disambiguation, strength-based association, and cross-reference between dosage forms and their detailed composition.
- **Level 4** represents the highest complexity, requiring the model to interpret *nested relationships* from descriptive, unstructured text. In **Section 6.5** (Nature and Contents of Container), a passage such as: “*White opaque HDPE bottle with a child-resistant polypropylene cap with an induction seal liner. Each pack contains 1 bottle with 180 g of powder and three measuring spoons. The green measuring spoon dispenses 100 mg. The blue measuring spoon dispenses 150 mg. The purple measuring spoon dispenses 1 g.”* must be parsed to identify the container types (e.g., bottle, measuring spoons), materials (e.g., HDPE, polypropylene), dosage values (e.g., 100 mg, 1 g), and the association between color, function, and capacity. This level requires the model to construct a deeply nested, semantically aligned structure from narrative text.

**Table 1:** The hierarchical organization of data elements aligned with the IDMP standards.

**Field**	**Section**	**Level 1**	**Level 2**	**Level 3**	**Level 4**
**1**	**1**	**Product Name**			
2		ATC Code			
3		Authorized Pharmaceutical Form			
4	**2**	Marketing Authorization Number			
5		Date Of First Authorization			
6		Date Of Latest Renewal			
7		Marketing Authorization Holder	Name		
8			Address		
9			Postal		
10			Country		
11	**3**	Therapeutic Indications Information			
12	**4**	Package	Package Description		
13			Package Composition	Container Description	
14				Container Type	
15				Package Item Material	
16				Package Component	Value
17					Component
					Material
18		Shelf Life	Value		
19			Special Precautions for Storage		
20	**5**	Composition Active	Active Substance Salt	Value	
21				Dosage	
22			Active Substance Base	Value	
23				Dosage	
24		Composition Excipient	Excipient		
25			Dosage		
26	**6**	Administrable Dose Form			
27		Unit Of Presentation			
28		Route Of Administration			

We believe this classification provides a useful framework for evaluating LLM-based systems in the efficient extraction of structured data from SmPCs.

The data extraction process uses the principles outlined in the EU IDMP Implementation Guide as follows:

1. Parsing mandatory conformance requirements: we parsed the mandatory conformance requirements specified in the EU IDMP Implementation Guide to identify essential fields.
2. Identification and extraction of mandatory fields: for each chapter in the guide, mandatory fields were identified and extracted to form the minimal configuration necessary to comply with regulatory standards.
3. Systematic capture of key fields: this approach ensured that all key fields essential for IDMP compliance were captured systematically.
4. Iterative review and mapping: an iterative process of reviewing each chapter's mandatory conformance alongside their interrelations led to the identification of a comprehensive set of minimal IDMP fields, which collectively constitute the core dataset of the EU IDMP data model.

The extraction model effectively breaks down the complex IDMP structure into well-defined fields, each representing critical aspects of the medicinal product information from regulatory data to packaging and composition. By systematically capturing data from regulatory documents such as SmPCs and structuring the extracted information into the IDMP fields, the model ensures accurate representation of medicinal products in accordance with regulatory requirements. The layered approach allows for clear differentiation between each IDMP section, from simple fields to intricate nested fields, providing a comprehensive yet precise framework for data extraction, which meets compliance requirements.

Complex SmPCs with multiple dosage forms and multiple packaging configurations present significant challenges due to their volatile structure. Such SmPCs require intricate management, as each dosage form or packaging type may have unique attributes, indications, administration routes, and safety information. This complexity can lead to increased variability and challenges in standardizing data across forms, making it difficult to conduct streamlined analyses or comparisons.

The nested nature of the elements in the IDMP model could present significant challenges for LLM when tasked with extracting data in the structured format expected by the IDMP framework. In free-form text, where explicit hierarchical markers are often absent, it might not be straightforward for an LLM to distinguish and reconstruct the precise nested structure required. For instance, given the text: “Aluminum/Aluminum blister with laminated desiccant (calcium oxide) containing 10 tablets. Each pack contains 20 or 40 film-coated tablets. Not all pack sizes may be marketed,” an LLM could identify components like “Aluminum/Aluminum blister” and “laminated desiccant (calcium oxide),” but it might struggle to infer and represent the hierarchical relationships accurately. The model would need to recognize that “blister” is a packaging type, “laminated desiccant” is a component of the blister, and “Aluminum/Aluminum” is the material used for the blister. Additionally, it could encounter difficulties in replicating and organizing this structure across variations in pack sizes (20 or 40 tablets). The implicit nature of such text might also hinder the model's ability to establish dependencies, such as linking pack sizes with their respective materials. If these challenges were not addressed, the limitations in interpreting semantic nuances, reconstructing hierarchical relationships, and mapping dependencies could prevent the accurate alignment of free-form descriptions with the deeply nested expectations of the IDMP model.

### 3.2 Data collection

We collected a dataset comprising 81 SmPC documents sourced from the European Medicines Agency (EMA) website. These documents represent a diverse array of medicinal products, ensuring a comprehensive sample for analysis. The data collection process involved selecting a representative mix of products in various therapeutic areas, dosage forms, and administration routes while excluding SmPCs that described multiple products or dosage forms to maintain consistency. We chose to subset the data to include simpler SmPCs with single dosage forms and packaging types, as this choice enables easier and more accurate information extraction by large language models. Working with simpler SmPCs allows us to control for variability, ensuring more consistent data interpretation and reliable findings, which can be crucial for generating robust and actionable insights. The full set of SmPC PDF documents used in the study is available at the following GitLab repository.^1^

The initial data extraction was conducted using the Large Language Model Claude 3 Sonnet^2^ with simple prompts and formatting constraints, establishing a baseline dataset. To validate and correct the data, a team of regulatory affairs specialists with expertise in SmPC interpretation manually reviewed each extracted element. The experts compared the LLM output with the original SmPC PDFs to identify and correct errors such as misclassified fields, incomplete values, or inconsistent terminology. A structured review protocol was followed, incorporating predefined annotation guidelines and decision rules for common ambiguity cases (e.g., distinguishing salt vs. base forms, resolving packaging hierarchies). Each SmPC was reviewed by at least one expert, and unclear cases were discussed collaboratively to ensure consistency. This expert-validated dataset served as the reference standard for evaluating the performance of our extraction models.

The final dataset comprised 5, 224 elements across key sections: 243 entries for Medicinal Product, 729 for Marketing Authorization Information, 81 for Therapeutic Indications, 1, 906 for Packaged Medicinal Product, 1, 538 for Ingredients, and 243 for Pharmaceutical Product. The extraction process presented several challenges. Distinguishing between salt and base forms of active substances was difficult when the LLM did not clearly specify them, necessitating reference to regulatory guidelines for accurate classification. Additionally, interpreting container descriptions and accurately identifying packaging compositions required strict adherence to EMA guidelines. Some fields, such as ATC Codes and specific excipient dosages, were frequently missing or misclassified, and discrepancies in Marketing Authorization Holder details, like partial address matches, required manual corrections.

### 3.3 Methodology

**Figure 1:**
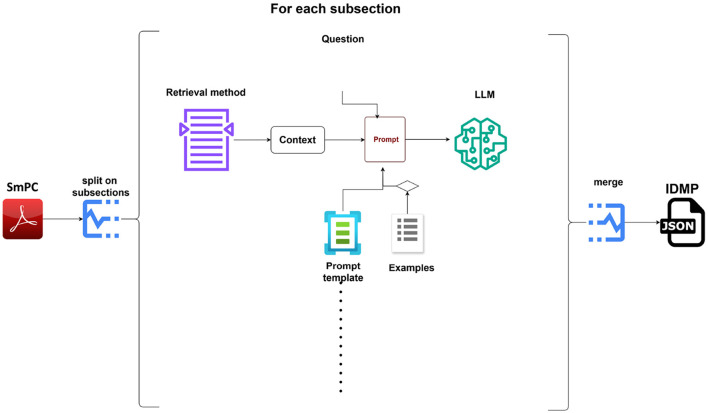
Workflow for extracting and structuring medicinal product data from SmPC to IDMP JSON.

To address our proposed scientific questions, we designed an experimental plan as follows : for each information field, we formulated a specific question and extracted relevant context from the SmPC using either a semantic similarity approach or a rule-based method. This context, along with the question, was then incorporated into a prompt template. If needed, examples were added to the prompt template, sourced from the EU IDMP Implementation Guide. The complete prompt was sent to the LLM, which processed it to retrieve the information in the correct format. Finally, the IDMP was reconstructed by assembling all extracted information into a JSON structure. The Figure 1 illustrates the pipeline for converting SmPC documents into structured IDMP-compliant JSON. The SmPC is segmented into subsections, and each subsection is processed using a retrieval method to extract context. Questions generated for each context are fed into a LLM via a prompt template, with optional example-based guidance. The outputs are merged to form a complete IDMP JSON representation of the medicinal product.

### 3.4 Retrieval strategy

#### 3.4.1 Semantic search-based method

**Figure 2:**
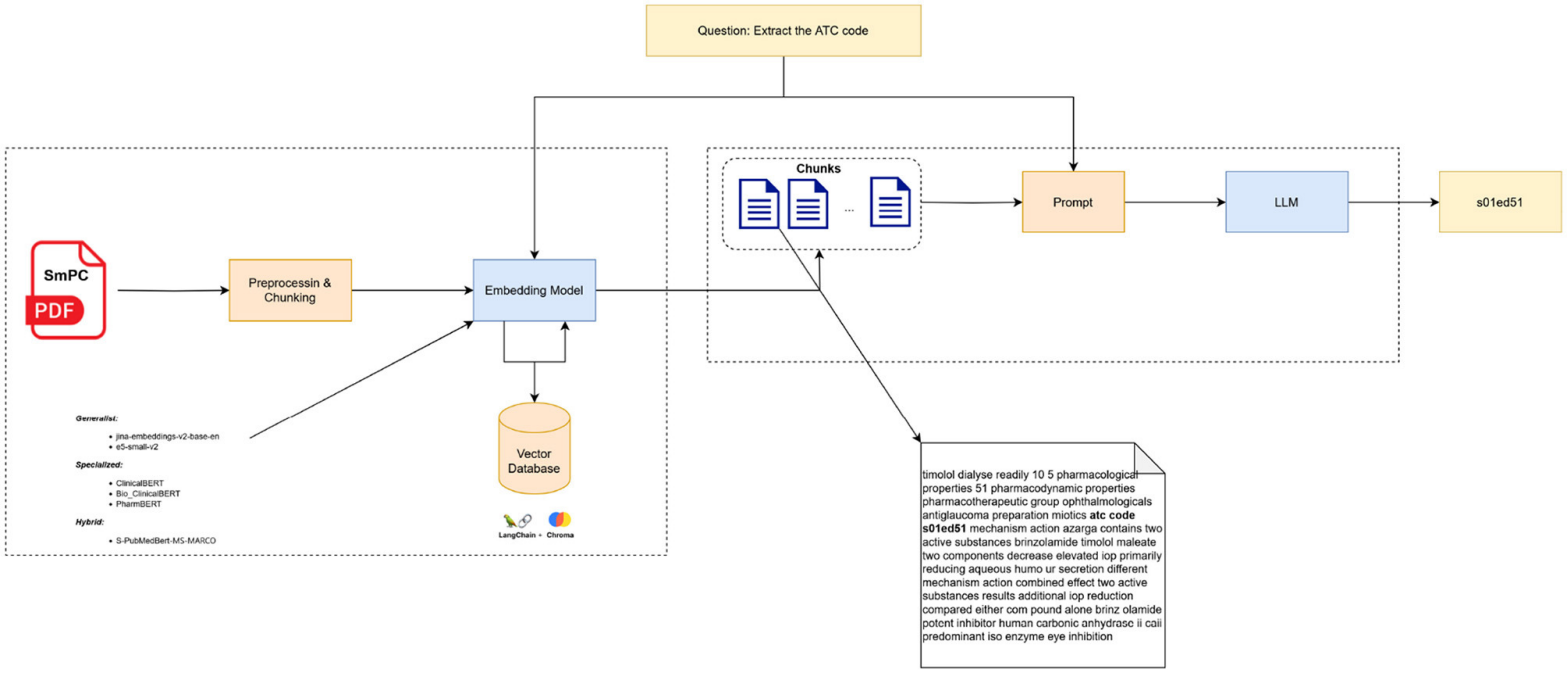
A RAG-based workflow leveraging semantic search with embedding models for extracting IDMP elements from SmPC documents.

For each field in the minimal IDMP structure, we aim to retrieve relevant information from a specific section, paragraph, or chunk of the SmPC. To achieve this, we employed a Retrieval-Augmented Generation strategy, which involves two key components: retrieval and generation. In the retrieval phase, each SmPC is split into chunks, which are stored in an embedding database for efficient querying (Figure 2).

We chose a chunk size of 500 characters with a 200-character overlap after empirical testing to balance context retention and minimize redundancy. During the retrieval phase, we used ChromaDB^3^ to store the chunk embeddings and applied cosine similarity to rank the relevance of the top *k* chunks (*k* = 20), ensuring that the most pertinent sections were retrieved for LLM processing.

We experimented with generalist, specialized, and hybrid clinical models:

- **Generalist models:** jina-embeddings-v2-base-en (33) and e5-small-v2 (34): trained on a variety of text types, these models demonstrate strong performance across a wide range of domains.
- **Specialized models:** ClinicalBERT (35) and Bio_ ClinicalBERT (36) offer deep insight into clinical and biomedical texts). PharmBERT trained on drug labeling documents (37).
- **Hybrid model:** S-PubMedBERT-MS-MARCO (38) fine-tuned on both clinical and generalist datasets. Versatile for mixed-domain tasks.

This systematic approach, combining chunking, embedding, and Semantic Search-based retrieval, ensures that the LLM has access to the most relevant sections of the SmPC, thereby improving the precision of information extraction for each IDMP element. The Figure 2 depicts the Semantic Search-based strategy used to extract relevant sections of SmPC for IDMP data.

#### 3.4.2 Rule-based method

Our second retrieval strategy involves creating a predefined mapping between the IDMP structure and specific chapters of the SmPC. For each IDMP field, we formulated targeted questions linked to specific sections within the SmPC. For example:

- Product Name: Mapped to SmPC Chapter 1. Name of the medicinal product
- ATC Code: Mapped to SmPC Chapter 5. Pharmacological properties
- Authorized Pharmaceutical Form: Mapped to SmPC Chapter 3. Pharmaceutical form
- Country/Continent of Marketing Authorization: Mapped to SmPC Chapter 8. Marketing authorization number(s)
- Date of First Authorization: Mapped to SmPC Chapter 9. Date of first authorization/renewal of the authorization
- Therapeutic Indication: Mapped to SmPC Chapter 4. Clinical particulars.

**Figure 3:**
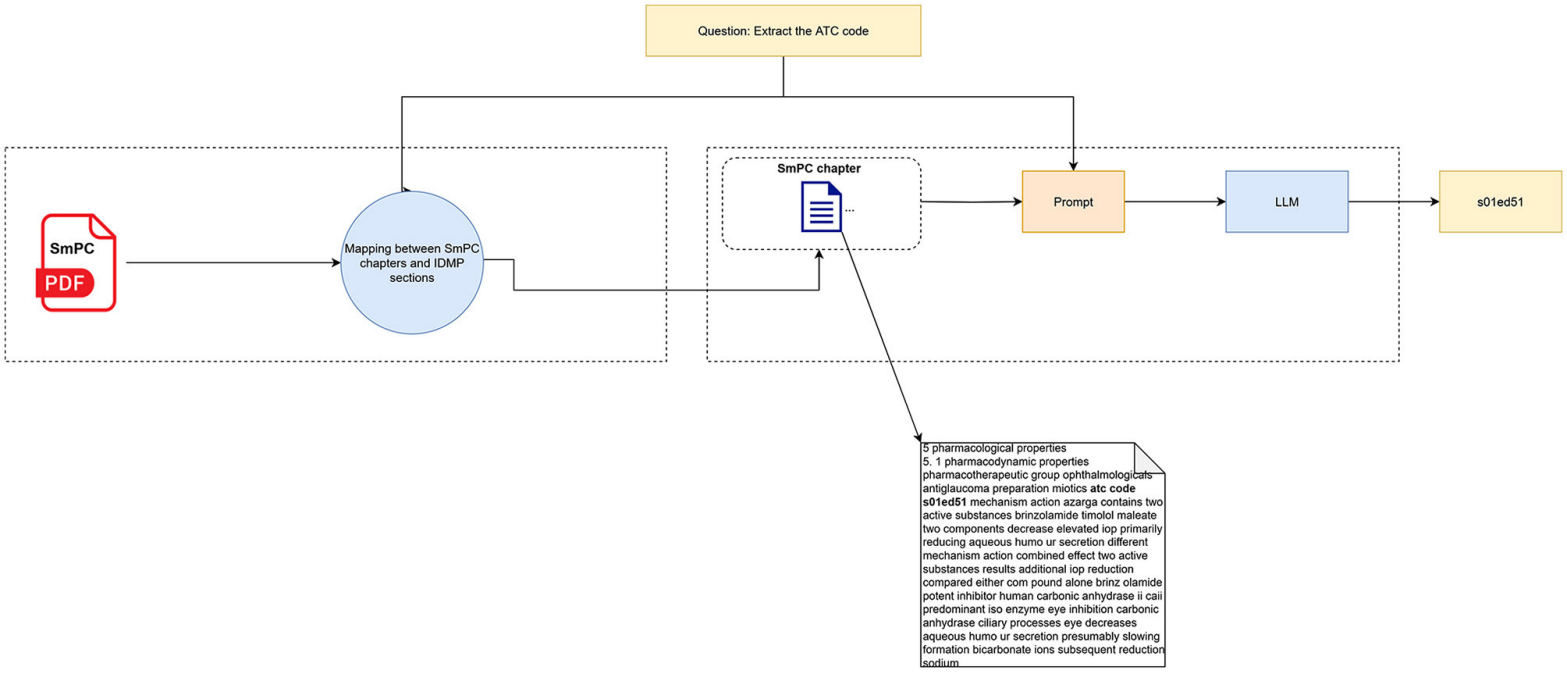
RAG-based workflow for extracting IDMP elements from SmPCs using rule-based retrieval.

By aligning each IDMP field with its corresponding SmPC chapter, we ensure accurate retrieval of relevant text during the information extraction process. This mapping relies on the EMA guidelines for structuring SmPCs, as cited above, providing a reliable framework for associating SmPC chapters with specific IDMP data points (Figure 3).

### 3.5 Prompts selection

In extracting data from SmPC documents to populate IDMP data objects, we employed three manual, few-shot prefix prompting strategies: Context-Action-Result-Example (CARE), Role-Input-Steps-Expectation (RISE), and a hybrid model Context- Input-Action-Expectation-Example (CIAEE). These strategies were carefully designed to potentially enhance the LLMs ability to accurately extract and structure complex medical information.

The CARE pattern is a manual, few-shot prefix prompt that may excel in scenarios requiring deep contextual understanding. By establishing the context, defining specific actions, and providing illustrative examples, CARE could significantly improve the accuracy of data extraction. However, the creation of effective examples might demand substantial time and expertise (19). To address this, we utilized examples provided by the EU IDMP Implementation Guide (20), which may ensure consistency and reduce the manual effort required in prompt design. Conversely, the RISE pattern emphasizes defining the input to be processed, outlining the procedural steps, and specifying the expected outcome. This structured approach might be particularly effective for complex extraction tasks, providing a clear pathway from input to output. Nevertheless, its detailed setup could potentially impede efficiency in dynamic environments where data is frequently changing (19).

To leverage the strengths of both CARE and RISE, we developed the CIAEE model, a hybrid prompt that integrates contextual depth with structured guidance. CIAEE combines the contextual and example-driven elements of CARE with the procedural clarity of RISE, potentially offering both precision and flexibility for more effective data extraction.

### 3.6 LLM selection

We selected Claude 3.5 Sonnet by Anthropic and Gemini 1.5 Flash based on a balance of performance and cost-efficiency. Claude 3.5 Sonnet demonstrates superior performance in complex reasoning and knowledge-based tasks, and is accessible through reliable platforms like Amazon Bedrock. Meanwhile, Gemini 1.5 Flash offers significantly lower token costs, making it an economical choice for extensive usage. Both models are deployed via hosted cloud solutions, providing seamless integration without the need for dedicated infrastructure. Table 2 compares key attributes of the Gemini 1.5 Flash and Claude 3.5 Sonnet LLMs.

**Table 2:** Comparative overview selected LLMs.

**Attribute**	**Gemini 1.5 Flash**	**Claude 3.5 Sonnet**
**Provider**	**Google**	**Anthropic**
Input context window	1M tokens	200K tokens
Max output tokens	8192 tokens	4096 tokens
Release date	May 14th 2024	June 20th 2024
Knowledge cutoff	November 2023	April 2024
Open source	No	No
API providers	Google Cloud Vertex AI	Anthropic AWS Bedrock^*a*^

^*a*^Available at: https://docsbot.ai/models/compare/claude-3-5-sonnet-20240620/gemini-1-5-flash-001.

### 3.7 Evaluation methods

#### 3.7.1 Methodology

To ensure the accuracy and consistency of the extracted data, we implemented an automated validation and comparison process using advanced NLP techniques. Our validation approach comprises data structure normalization and similarity metrics computation. Firstly, we standardized variations in data formats to achieve consistency across different data sources, enabling accurate comparisons. Secondly, we applied a range of NLP-based similarity metrics to quantitatively assess the similarity between corresponding data fields.

#### 3.7.2 Data normalization

Robust data normalization processes were integral to our validation strategy, ensuring that data comparisons were meaningful and accurate.

##### 3.7.2.1 Handling delimiters and ranges

For fields such as marketing authorization numbers, we addressed variations due to different delimiters or range representations. For example, before normalization an authorization number like

[~EU/1/22/1646/001,~ ~EU/1/22/1646/002-004~]

would be expanded to include all numbers in the range, resulting in

[~EU/1/22/1646/001,~ ~EU/1/22/1646/002,~ ~EU/1/22/1646/003,~ ~EU/1/22/1646/004~].

This expansion ensures that every individual authorization number is explicitly represented for accurate comparison.

##### 3.7.2.2 Parsing JSON structures

Some data fields contained strings representing JSON lists (e.g., [“value1,” “value2”]). We identified such cases and converted these strings into actual list objects to maintain structural integrity. So, input string

'[~ingredientA,~ ~ingredientB~]'

after parsing becomes

[~ingredientA,~ ~ingredientB~]

##### 3.7.2.3 Ensuring uniqueness and cleanliness

We removed extraneous characters (such as brackets or quotes) and duplicate entries to ensure that the data was clean and that each item was unique. If we have

[~value1,~ ~value1,~ ~value2~]

cleaning will give

[~value1,~ ~value2~]

##### 3.7.2.4 Normalization of dictionary keys

A critical step in our approach was the normalization of all dictionary keys to lowercase using the normalize_dict_keys function. This recursive function ensures that key comparisons are case-insensitive, addressing potential mismatches caused by inconsistent capitalization. For example, original keys

~ProductName~: ~Aspirin,~ ~productname~: ~Aspirin,~ ~PRODUCTNAME~: ~Aspirin~

after normalization become

~productname”: ~Aspirin~.

##### 3.7.2.5 Special handling of default values and empty strings

Certain fields in the data structures may have default values or be empty, which can imply the same semantic meaning. For the field special_precautions_for_storage, an empty string is considered equivalent to the default phrase. Suppose that for the statement “*This medicinal product does not require any special storage conditions.”* there exist two structures

- Data Structure A: “special_precautions_for_storage”: “”
- Data Structure B: “special_precautions_for_storage”: “This medicinal product does not require any special storage conditions.”

In this case special handling gives

**Interpretation**: Both indicate no special storage precautions are needed. **Action**: Assign full similarity scores for this field.

In the field shelf_life.type.value, empty strings are treated as equivalent to standard shelf life values like “3 years” or “6 years”.

##### 3.7.2.6 Normalization and comparison of lists and sets

Data structures often contain lists of items where the order is not significant. To accurately compare such lists, we normalized and compared them as sets. It allows to introduce **set-based comparison**

- List A: [“lactose,” “sucrose”]
- List B: [“sucrose,” “lactose”]
- Metric Similarity = 1.0
- **Interpretation**: Perfect match regardless of order.

and **matching items in lists of dictionaries**

Ingredient Lists:

- Structure A: [“name”: “aspirin,” “quantity”: “500mg”, “name”: “caffeine,” “quantity”: “50mg”]
- Structure B: [“name”: “caffeine,” “quantity”: “50mg”, “name”: “aspirin,” “quantity”: “500mg”]

Matching Criteria: Normalize and compare the “name” field.

Result: Items are matched correctly despite different ordering.

##### 3.7.2.7 Date field handling

For fields representing dates, listed in DATE_FIELDS, we parsed the strings into date objects using the parse_date function and compared the dates. It can be illustrated as follows

- Structure A: “date_of_first_authorization”: “2021-05-01”
- Structure B: “date_of_first_authorization”: “May 1, 2021”

**After parsing**: both are recognized as the same date.

**Action**: assign full similarity scores.

#### 3.7.3 Similarity metrics and statistics

We utilized a variety of NLP-based metrics to evaluate data similarity, as each provides a different perspective on how well the extracted content aligns with the reference data. No single metric serves as a definitive reference; rather, these metrics are complementary, allowing for a more robust and nuanced assessment of model performance across lexical, structural, and semantic dimensions.

- **Exact match**: evaluates whether fields match exactly.
- **Jaccard similarity**: measures the overlap between sets of tokens (39).
- **Levenshtein similarity**: computes character-level similarity based on edit distance (39).
- **Cosine similarity (token-based)**: measures the cosine of the angle between TF-IDF text vectors (39).
- **BERT-based similarity (BERTScore)**: captures semantic similarity using contextual embeddings from pretrained transformer models (40).
- **BLEU score**: focuses on the precision of n-gram overlaps, commonly used in machine translation (41).
- **ROUGE scores**: recall-oriented measures capturing n-gram overlaps (42).
- **METEOR score**: balances precision and recall with synonym matching and stemming for unigram matches (43).

To assess the statistical significance of various experimental parameters, such as the language model, prompt design, embedding model, and retrieval technique, on these similarity metrics, we applied non-parametric Kruskal–Wallis tests, which are appropriate for right-skewed or non-normally distributed data (44).

## 4 Results

This section presents the results of our experiments. We begin with a general comparison to identify the key elements that influence the quality of extraction. In the next step, we analyze each component LLM, prompt designs, embedding models, and retrieval techniques in detail to understand its specific impact on performance.

**Figure 4:**
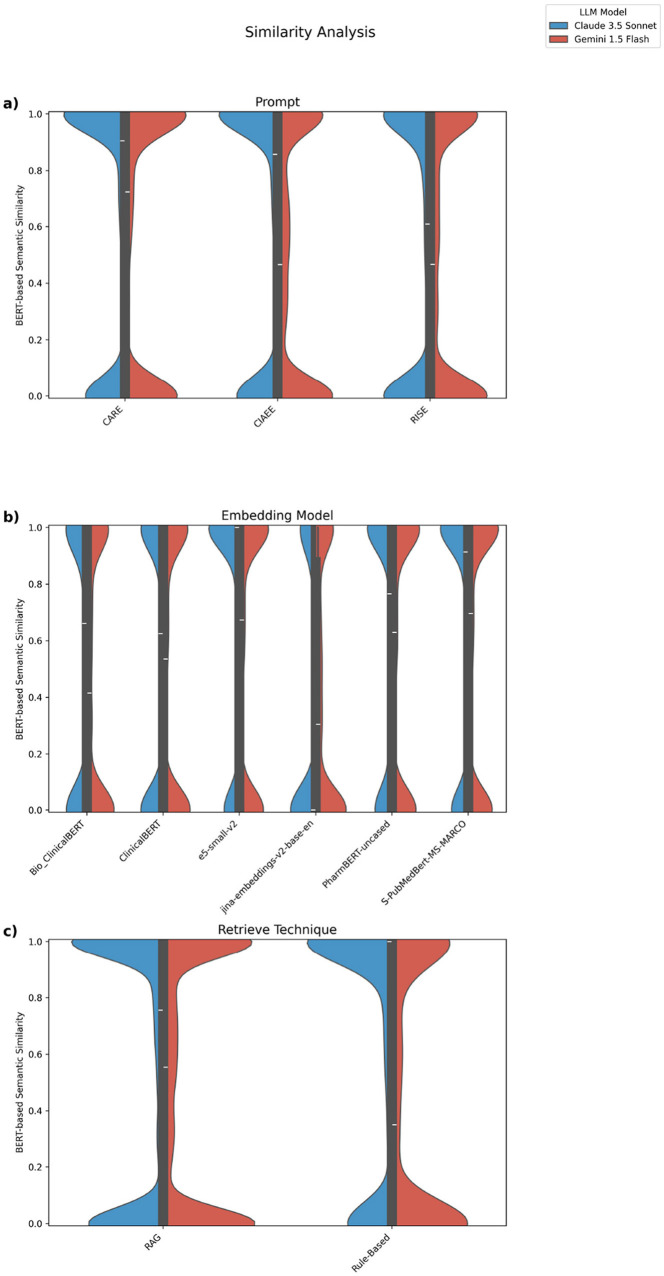
Comparative analysis of semantic similarity scores across multiple experimental factors using the BERT-based semantic similarity metric from the SentenceTransformer model (all-MiniLM-L6-v2). **(a)** Distribution of similarity scores by prompt. **(b)** Distribution by embedding model. **(c)** Distribution by retrieval technique.

Figure 4 presents a comparative analysis of semantic similarity scores, measured using the BERT-based semantic similarity metric from the SentenceTransformer model ('all-MiniLM-L6-v2'). The analysis is grouped by LLM (Claude3.5 Sonnet and Gemini 1.5 Flash), with each model represented by a distinct color on the violin plots. Each violin plot illustrates the spread and density of similarity scores, showcasing how prompt templates, embedding models, retrieval techniques, and embedding types impact the distribution of these scores across the different LLM.

The analysis of semantic similarity results reveals a bimodal distribution, with scores grouped around two distinct values: one close to 1.0, indicating high similarity, and the other close to 0, suggesting low similarity. The primary reason for the bimodal distribution in semantic similarity scores appears to stem from our prompt design. In the RAG approach, we explicitly instructed the LLM to return an empty string if it found no relevant information in the retrieved context. This design choice naturally produces low similarity scores clustering around 0, as the model signals an absence of relevant data whenever it fails to identify useful information. However, the rule-based retrieval approach is designed to ensure that relevant information is present in the context. Here, we direct the LLM to extract information from a specific section where we are certain it can be found. However, despite this controlled setup, we still observe instances of low similarity scores (near zero), which can only be explained by the model's inability to effectively extract the targeted information, even when it is clearly available.

Furthermore, a clear difference in performance between the models is evident: Gemini 1.5 Flash generally produces lower similarity scores than Claude 3.5 Sonnet, particularly in the rule-based retrieval context. This distinction is most pronounced in the rule-based retrieval plot, where Gemini's similarity scores tend to cluster more frequently at the lower end compared to those of Claude. Our analysis shows that the CARE prompt consistently outperforms other prompt designs, followed by CIAEE and then RISE. It appears that CARE and CIAEE utilize examples, which effectively guide the large language models to produce more accurate and contextually appropriate responses (Figure 4).

When evaluating embedding models, the e5-small-v2 model proves to be the most efficient, followed by S-PubMedBERT-MS-MARCO, PharmBERT, ClinicalBERT, Bio_ClinicalBERT, and finally, jina-embeddings-v2-base-en. In comparing the performance of the LLM, Claude 3.5 Sonnet consistently delivers better results than Gemini 1.5 Flash across most embedding models. An exception to this trend is observed with jina-embeddings-v2-base-en, where Gemini performs better. Additionally, our findings indicate that the rule-based retrieval technique outperforms the Retrieval-Augmented Generation approach on average. This superiority likely arises from the rule-based method's ability to consistently provide relevant context, enabling more precise information extraction by the LLM.

We discuss some limitations to rule-based approaches below. In addition to our primary analysis, we sought to determine the statistical significance of each parameter's effect on extraction performance namely, the impact of LLMs, prompt designs, embedding models, and retrieval techniques (Table 3). Given that the data did not follow a normal distribution, we applied the non-parametric Kruskal-Wallis test for comparing medians across metrics.

**Table 3:** Statistical comparison of metrics across LLMs, prompt designs, embedding types, and retrieval techniques.

**Parameter**	**Metric**	**H-statistic**
LLM	ANLS	594.22
	BERT score F1	606.86
	BLEU score	595.83
	METEOR score	485.50
	ROUGE score	680.37
Prompt	ANLS	516.24
	BERT score F1	420.76
	BLEU score	556.68
	METEOR score	420.89
	ROUGE score	609.94
Embedding model	ANLS	230.25
	BERT score F1	221.58
	BLEU score	188.17
	METEOR score	182.34
	ROUGE score	247.67
Retrieve technique	ANLS	51.67
	BERT score F1	51.30
	BLEU score	59.69
	METEOR score	54.40
	ROUGE score	79.31

This table presents the H-statistics from Kruskal–Wallis tests evaluating the impact of four factors, LLM, prompt design, embedding type, and retrieval technique, on five performance metrics: ANLS, BERT Score F1, BLEU Score, METEOR Score, and ROUGE Score. All tests indicate statistically significant differences in distributions across groups, with *p*-values < 0.001.

These results underscore that each parameter LLM, prompt design, embedding type, and retrieval technique has a statistically significant impact on the various performance metrics, with all *p*-values being less than 0.001 (*p* < 0.001). Notably, LLMs and prompt designs show the highest H statistics across multiple metrics, highlighting their substantial influence on both structural and semantic similarity.

### 4.1 Performance of prompt structures

**Figure 5:**
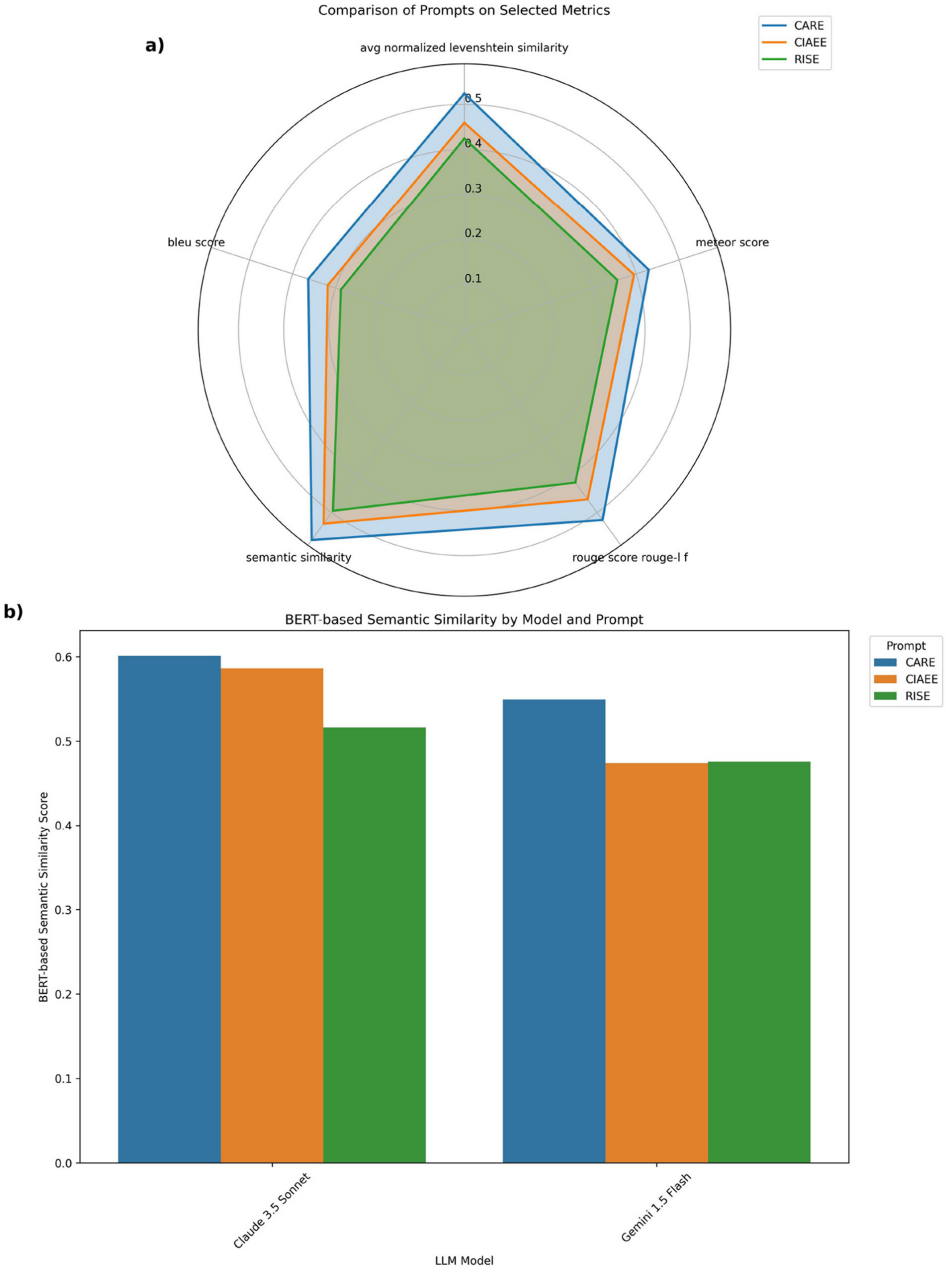
**(a)** Radar chart comparing the average performance of different prompts across multiple similarity metrics. **(b)** Grouped bar plot showing the average BERT-based semantic similarity scores achieved by each LLM across different prompts.

Now we will discuss the impact of prompt structure on model performance, as illustrated in the two plots (Figure 5).

The radar chart compares the performance of the CARE, CIAEE, and RISE prompts across key similarity metrics (e.g., BERT score F1, ROUGE Score, SBERT similarity). CARE shows the largest area, indicating superior alignment with reference text across metrics. This suggests that CARE's example-based structure provides clearer guidance for the LLM, resulting in more accurate, contextually relevant responses. CIAEE, though effective, lags slightly behind CARE, while RISE consistently ranks the lowest, highlighting the advantage of prompts with more structured guidance. The bar chart further supports these findings, comparing semantic similarity scores for each prompt across two LLM, Claude 3.5 Sonnet and Gemini 1.5 Flash. CARE achieves the highest scores on both models, with Claude 3.5 Sonnet generally outperforming Gemini 1.5 Flash. This modest model difference underscores that while model selection matters, prompt design particularly example-based prompts like CARE plays a more substantial role in achieving high semantic similarity.

### 4.2 Section-wise performance analysis

We conducted a section-wise performance analysis across different parts of the minimal IDMP data model, focusing on how embedding models and prompt structures affect semantic similarity in various sections such as therapeutic indications, product composition, and packaging. This detailed breakdown helps us understand the strengths and weaknesses of each approach in capturing domain-specific semantic information.

**Figure 6:**
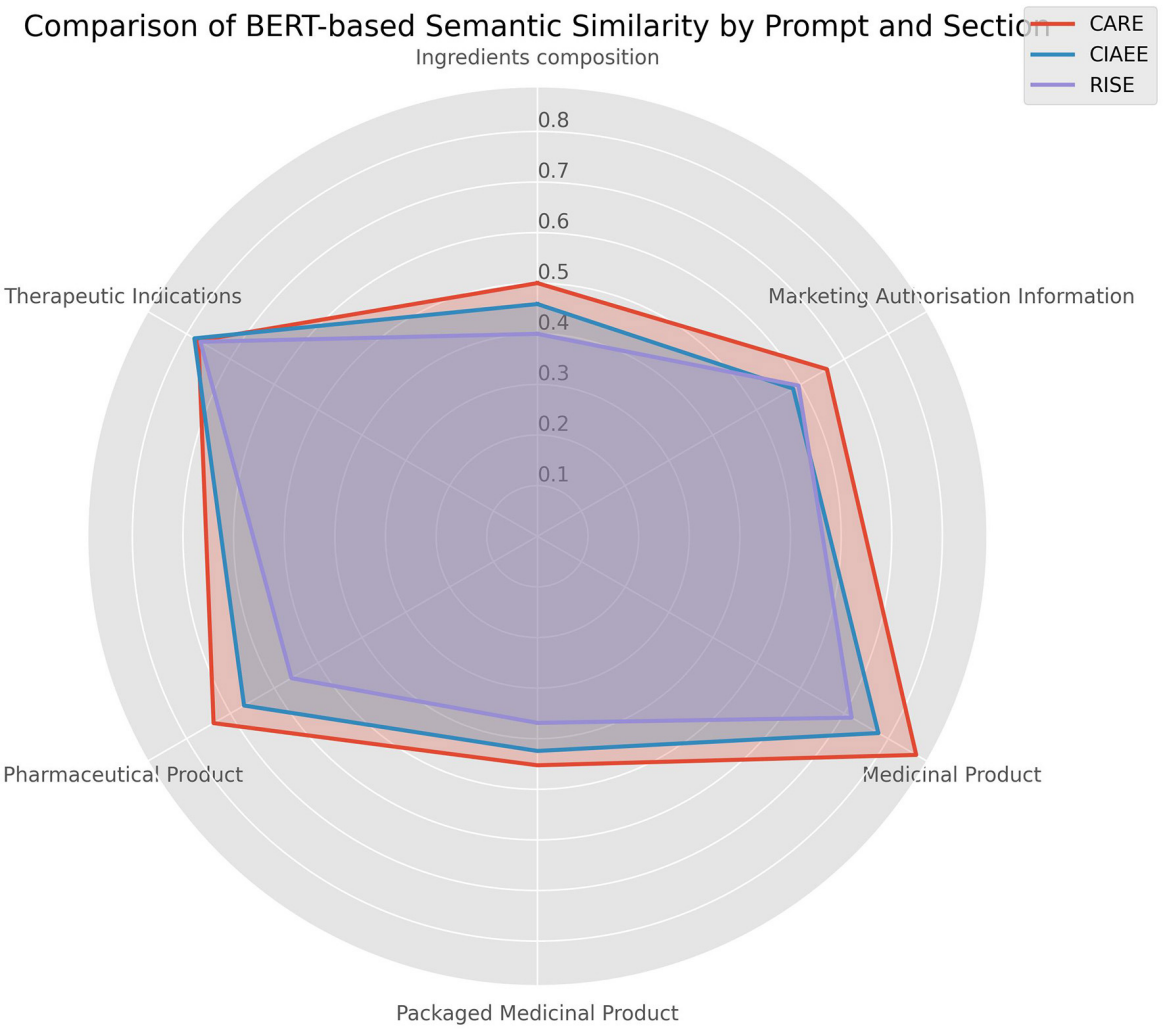
Section-wise performance analysis of BERT-based semantic similarity across IDMP model sections.

The radar chart provides a comparative view of semantic similarity scores by section across different prompt types (CARE, CIAEE, and RISE) within the IDMP model (Figure 6). This visualization allows us to observe how prompt design impacts performance across sections. CARE consistently shows the largest area on the radar chart, followed by CIAEE, with RISE trailing. This reinforces earlier findings that CARE's example-based approach leads to higher semantic similarity across all IDMP sections, enhancing response accuracy and relevance. Semantic similarity scores vary by section, with “Marketing Authorization Information” and “Therapeutic Indications” showing higher scores, while sections like “Ingredients composition” and “Packaged Medicinal Product” have lower scores. This variation suggests that certain sections may inherently support more straightforward semantic alignment, possibly due to less complex language or more explicit structure. The radar chart highlights the interplay between LLM, prompt type, and section. The CARE prompt, in particular, achieves higher similarity across most sections, demonstrating its effectiveness in guiding the LLM to align better with the IDMP model's structure and content.

**Figure 7:**
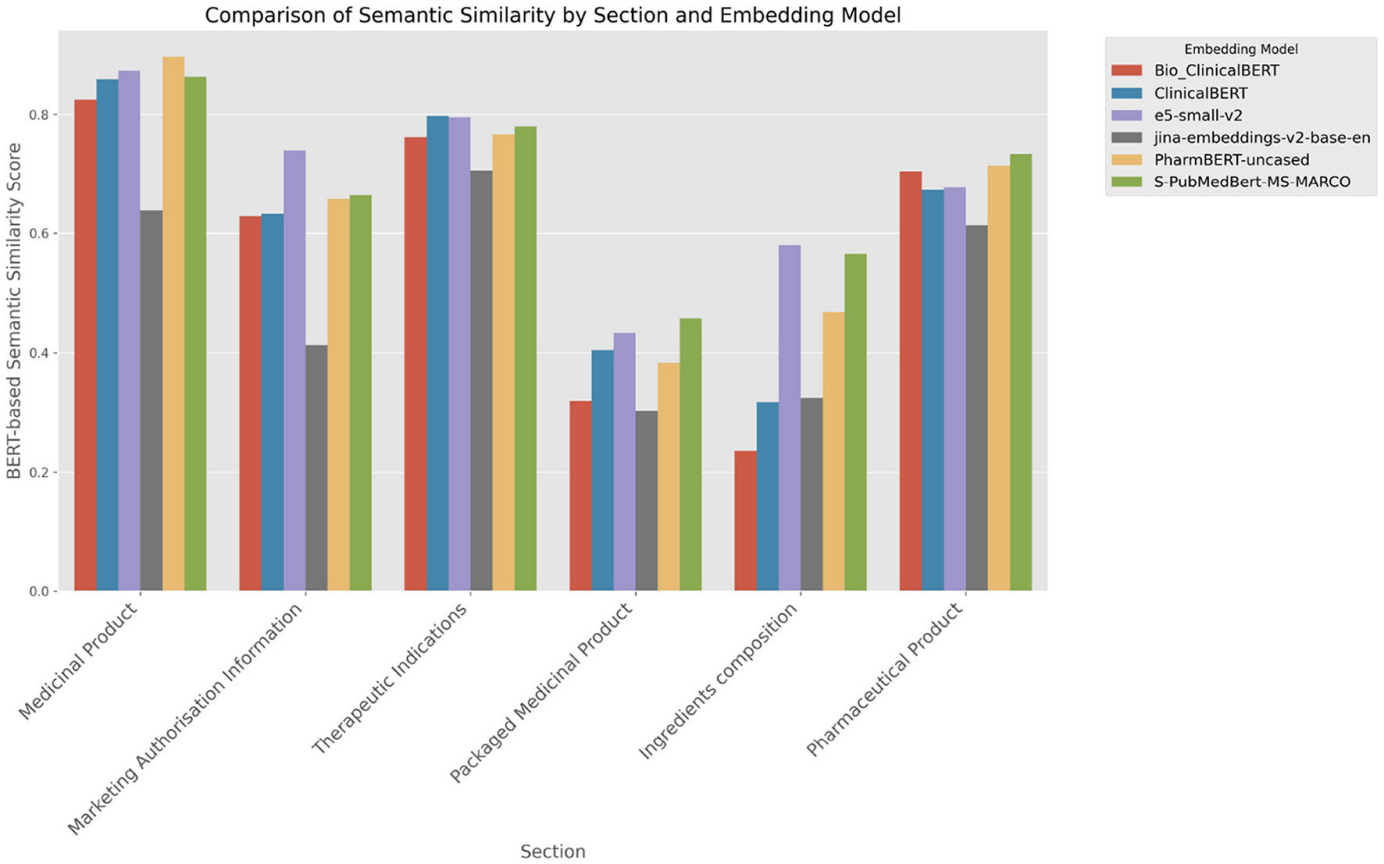
Performance of embedding models across IDMP data model sections.

The bar chart in Figure 7 presents a comparative evaluation of embedding model performance across different sections of the IDMP data model, using BERT-based semantic similarity. PharmBERT-uncased consistently performs well, particularly in the Medicinal Product and Pharmaceutical Product sections. e5-small-v2, ClinicalBERT, S-PubMedBERT-MS-MARCO, and Bio_ClinicalBERT also achieve strong scores across most sections. In contrast, jina-embeddings-v2-base-en shows the lowest performance throughout.

For specific sections:

- e5-small-v2 achieves the highest scores in Marketing Authorization Information and Therapeutic Indications.
- S-PubMedBERT-MS-MARCO performs best in Packaged Medicinal Product and is among the top models in Ingredient Composition.
- PharmBERT-uncased is among the most consistent top performers overall.

These results highlight the section-specific variability in performance across embedding models, underscoring the importance of selecting embeddings tailored to the structure and content of each regulatory section.

### 4.3 Comparison of semantic search and rule-based methods

**Figure 8:**
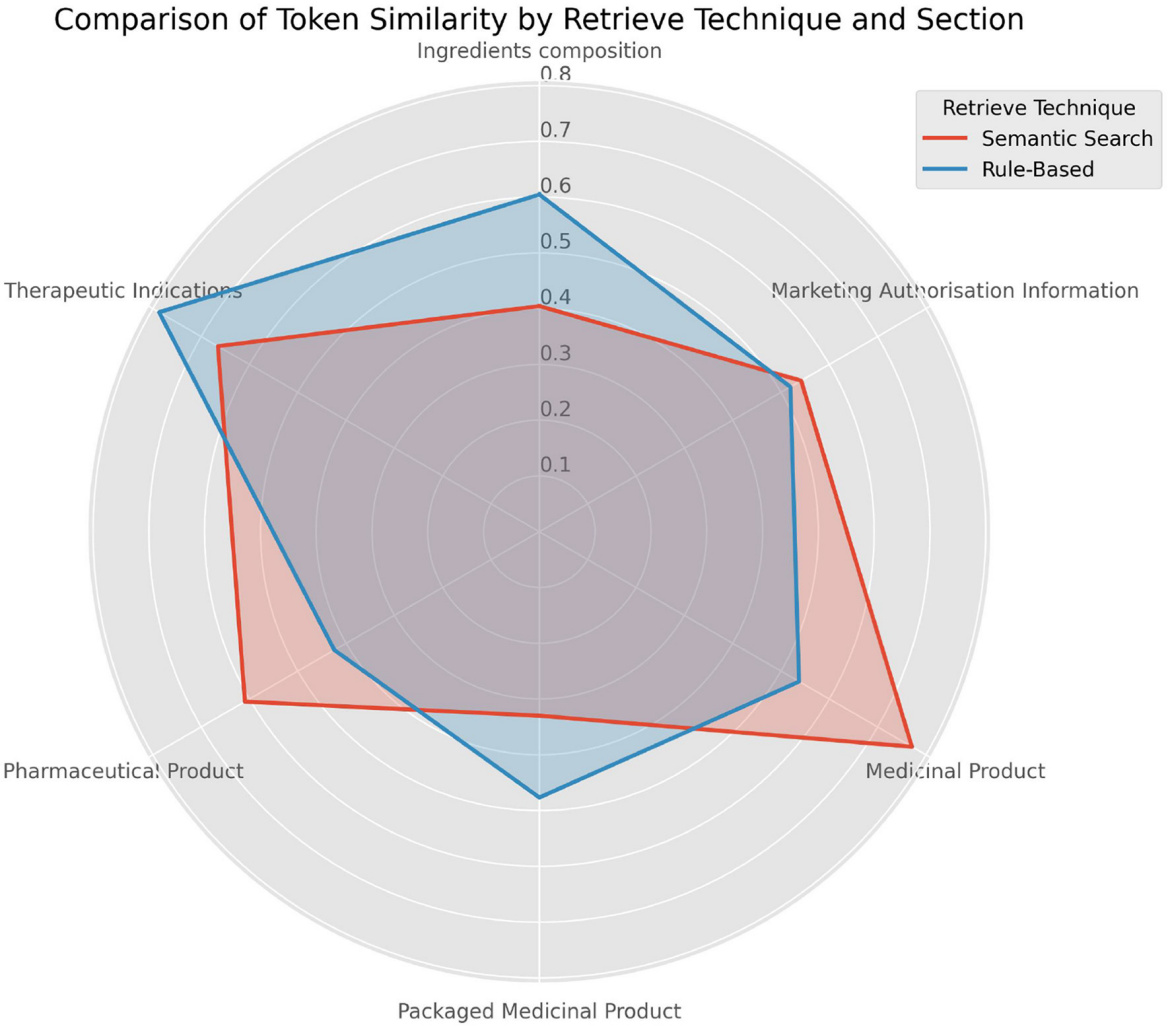
Comparison of semantic search and rule-based methods across IDMP data model sections. The radar plot illustrates average token-level similarity scores for each retrieval method across different IDMP sections, highlighting performance variations between semantic and rule-based approaches.

To assess the effectiveness of different retrieval techniques in achieving semantic alignment within the IDMP data model, we compared Semantic Search and Rule-Based approaches. The radar chart in Figure 8 illustrates the average token-level similarity scores across key IDMP sections: Therapeutic Indications, Marketing Authorization Information, Ingredients Composition, Pharmaceutical Product, Packaged Medicinal Product, and Medicinal Product. This comparison highlights the relative strengths of each method in capturing domain-specific content in regulatory contexts.

The Rule-Based approach demonstrates stronger performance in three sections, particularly Therapeutic Indications and Ingredients Composition, where it achieves scores of ~0.8 and 0.6, respectively. Conversely, Semantic Search outperforms in sections like Medicinal Product (0.76) and Pharmaceutical Product (0.51), suggesting better alignment in those areas. In Marketing Authorization Information, both techniques perform similarly, with scores around 0.51 (Rule-Based) and 0.53 (Semantic Search). For Packaged Medicinal Product, Rule-Based again leads with a score of 0.48 vs. 0.32 for Semantic Search.

### 4.4 In-depth analysis

#### 4.4.1 Exact matching: “ATC Code” and “Marketing Authorization Number”

**Figure 9:**
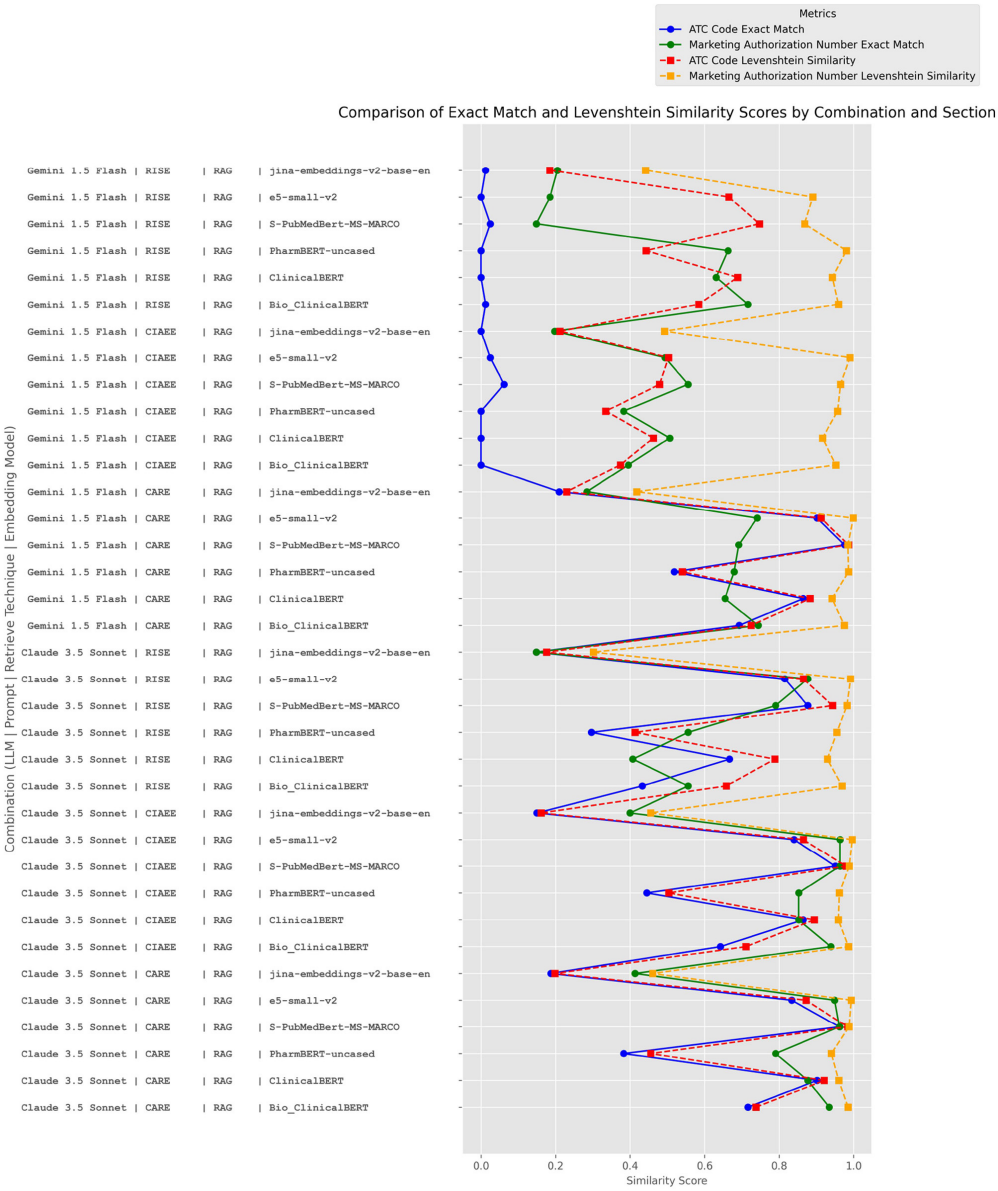
Exact matching performance for “ATC Code” and “Marketing Authorization Number” sections across configurations.

The plot (Figure 9) provides a detailed view of the average exact match proportions across various configurations, grouped by “ATC Code” and “Marketing Authorization Number” sections. For the ATC Code section, the top-performing configurations are dominated by combinations involving the Gemini 1.5 Flash and Claude 3.5 Sonnet models, especially when paired with the S-PubMedBert-MS-MARCO and ClinicalBERT embedding models. The highest exact match similarity score of 0.9753 was achieved by Gemini 1.5 Flash with the CARE prompt, Seamantic Search retrieval technique, and S-PubMedBert-MS-MARCO embedding.

Across configurations, a consistent trend appears where S-PubMedBert-MS-MARCO embedding enhances performance with both Gemini 1.5 Flash and Claude 3.5 Sonnet, achieving similarity scores above 0.9 in multiple instances. e5-small-v2 and Bio_ClinicalBERT also exhibit reliable performance, supporting the idea that domain-specific embeddings play a pivotal role in accurately capturing the semantics of ATC Code content. Interestingly, rule-based techniques, when combined with the CARE prompt (e.g., with Claude 3.5 Sonnet), reach relatively high scores (0.95), which implies that manually engineered rules can perform well under certain controlled contexts but may lack the flexibility seen in high-performing embedding-driven approaches.

The Marketing Authorization Number section presents slightly different trends. Here, Claude 3.5 Sonnet combined with CIAEE or CARE prompts and S-PubMedBert-MS-MARCO or e5-small-v2 embeddings achieves the highest similarity scores (0.96). This highlights a notable consistency, where S-PubMedBert-MS-MARCO again ranks among the best-performing embeddings, indicating its utility across diverse biomedical contexts.

However, e5-small-v2 also achieves top scores in this section, which may be due to its capacity for general-purpose embeddings that still provide some adaptability to structured numeric or alphanumeric identifiers, typical of authorization numbers. Unlike the ATC Code section, the Marketing Authorization Number section also sees Bio_ ClinicalBERT and ClinicalBERT perform effectively, suggesting that these embeddings offer a balanced approach for handling both structured and semi-structured content in regulatory contexts. Notably, rule-based retrieval techniques and simpler embeddings, such as jina-embeddings-v2-base-en, result in mid-to-low similarity scores. This outcome highlights the challenge of using generic or rule-based approaches in cases where identifier recognition and validation require nuanced context understanding.

#### 4.4.2 Therapeutic indication

**Figure 10:**
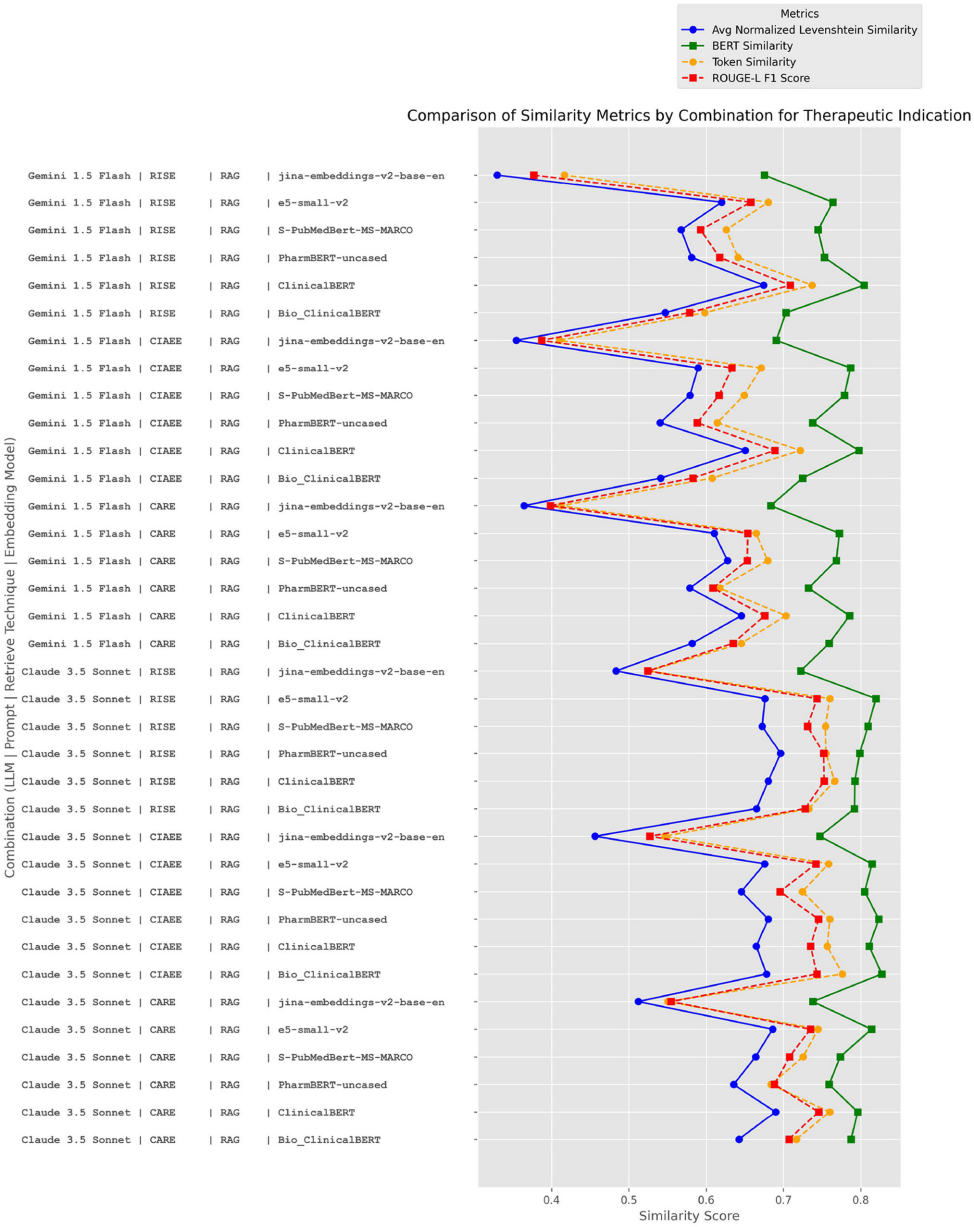
Performance comparison of LLM, prompt, retrieval technique, and embedding model combinations in the therapeutic indication section.

We will now delve into the details of the Therapeutic Indication section, comparing various combinations of Large Language Model (LLM), Prompt, Retrieval Technique, and Embedding Model based on two primary metrics: BERT Similarity and Token Similarity, as well as additional metrics like Average Normalized Levenshtein Similarity and ROUGE Score. This analysis aims to understand the differences in semantic retrieval quality vs. word-level retrieval quality, as well as the importance of the order of retrieved terms. The results of these four metrics are plotted on a graph (Figure 10), showing their values according to different combinations of LLM, Prompt, Retrieval Technique, and Embedding Model.

We observe that the Claude and Gemini models perform comparably, with a slight advantage for Claude. Additionally, the four metrics exhibit similar trends across combinations, with the semantic metric having the highest values, followed by token similarity (measured via TF-IDF), then ROUGE Score, and finally the Average Normalized Levenshtein Similarity. Interestingly, the prompt does not appear to be a determining factor in these results. However, when it comes to Embedding Models, we note that specialized models, such as BioClinicalBERT, ClinicalBERT, and PharmBERT, tend to perform better overall, with e5-small-v2 also remaining competitive. We also observe that the retrieved sequences closely align with the ground truth, showing minimal differences between token similarity and the ROUGE metric indicating that tokens are retrieved in the correct order. However, the models tend to retrieve more text than the ground truth, as reflected in the Average Normalized Levenshtein Similarity metric. The two models that optimize performance across these four metrics are, on average, ClinicalBERT and BioClinicalBERT. This can likely be attributed to their training on medical terms, which enhances their ability to handle domain-specific language.

#### 4.4.3 Extraction of date-type information

**Figure 11:**
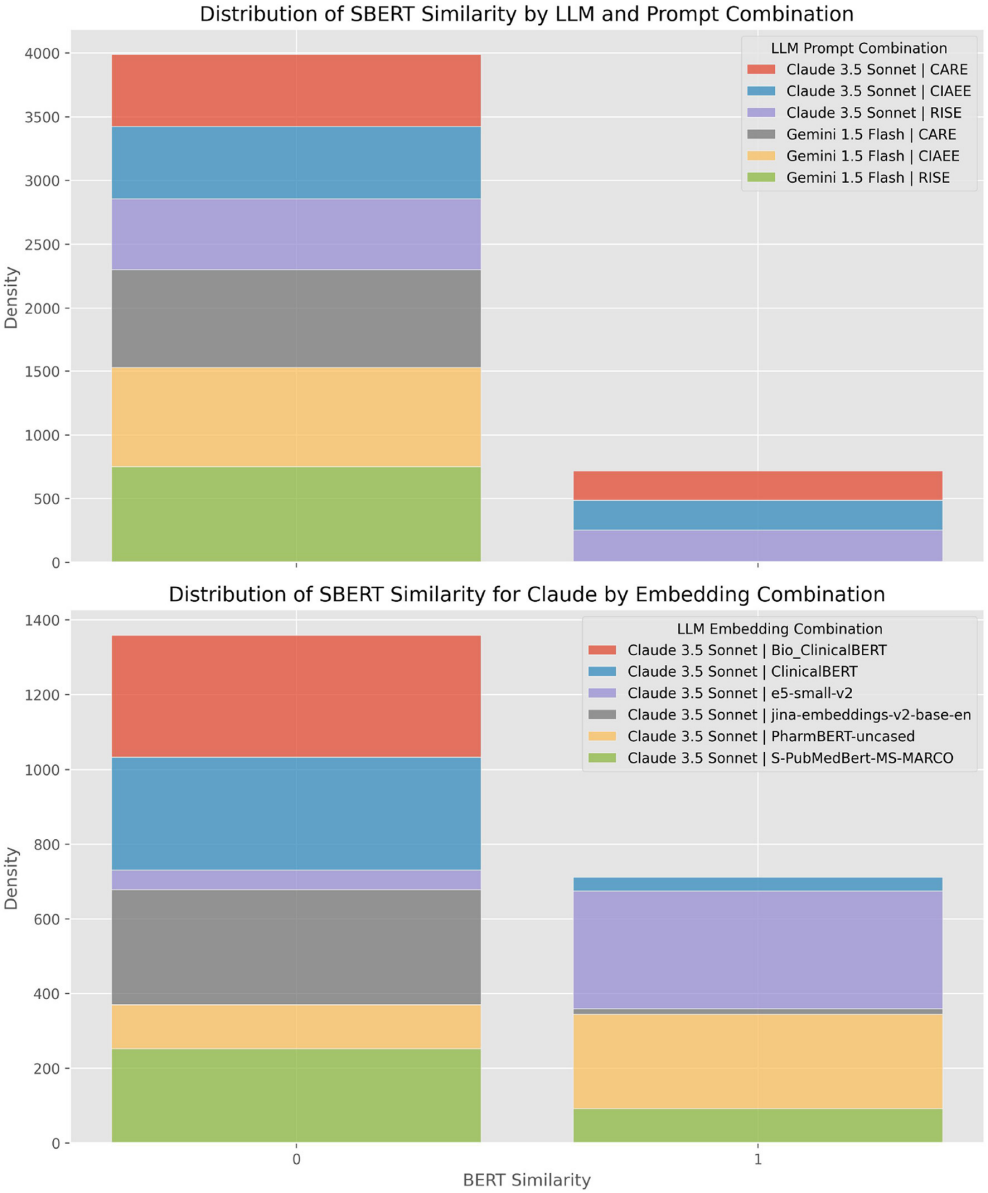
Distribution of Jaccard similarity for date extraction in “Date of First Authorization” and “Date of Latest Renewal” fields.

In the Figure 11, we compare the distribution of Jaccard similarity for dates found in two fields: Date of First Authorization and Date of Latest Renewal. We observe that, due to formatting corrections applied before metric measurements and prompt expectations, values are distributed between 0 and 1, indicating that the models are not hallucinating. However, we note that Gemini 1.5 Flash was unable to extract any dates. Additionally, we observe that the prompt has no impact on date extraction. In terms of embedding models, PharmBERT and e5-small-v2 achieved the highest number of correct extractions.

#### 4.4.4 Complex extraction level analysis

**Figure 12:**
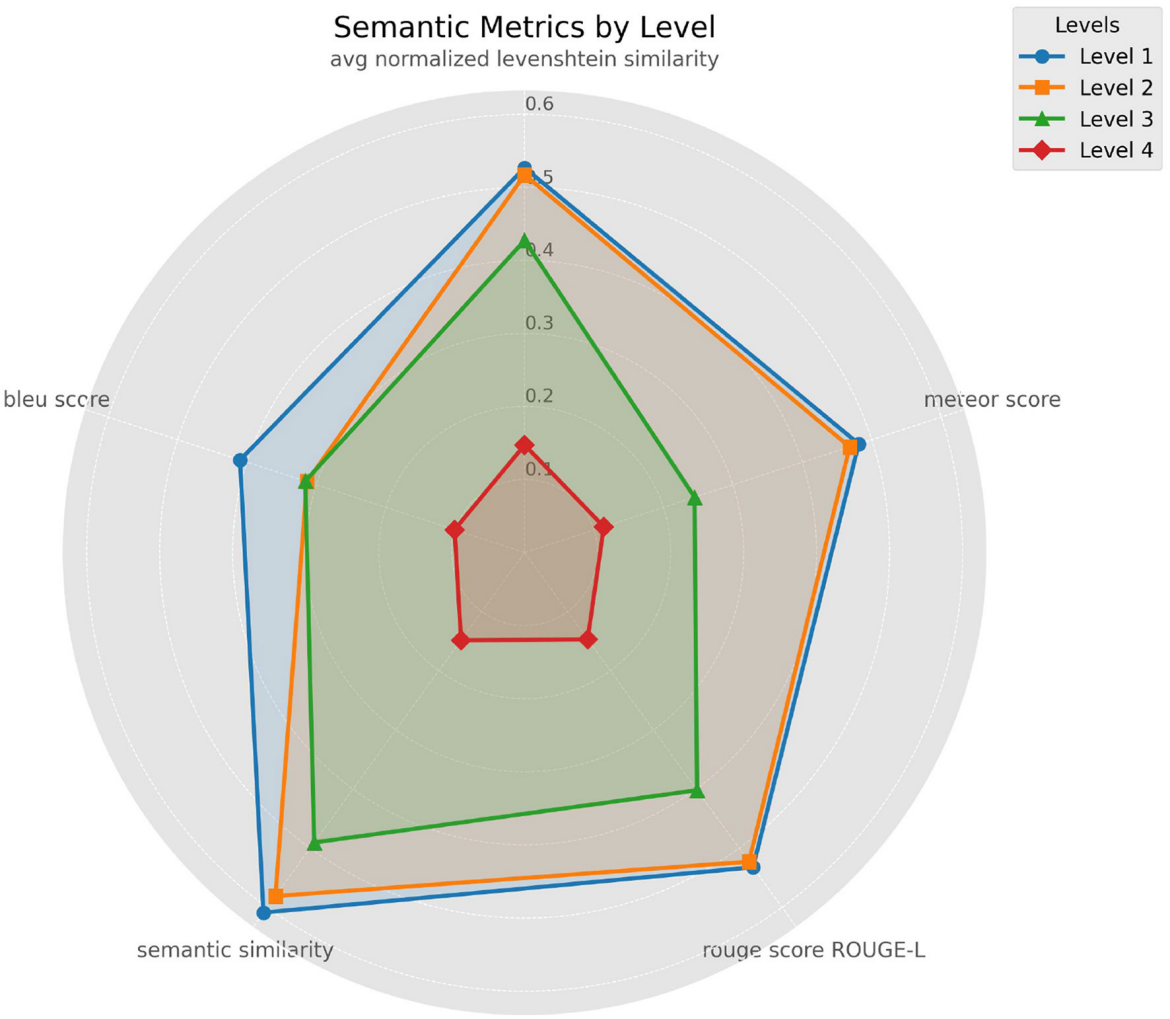
Data extraction quality across different levels of nesting.

We will now analyze the quality of data extraction based on the data level and degree of nesting, ranging from levels 1 to 4. At level 1, extraction is straightforward, such as retrieving the medicinal product name, as outlined in Table 1. This analysis considers various metrics, including average normalized Levenshtein similarity, BLEU, ROUGE Score, METEOR, and BERT score. The results are shown in the accompanying figure (Figure 12).

We observe that extraction at level 4 yields very low scores, averaging around 15%. Level 3 follows, with metrics ranging from ~0.3 for BLEU to 0.5 for BERT score. Levels 1 and 2 show comparable extraction quality, with the exception of BLEU, which appears highly sensitive. These results indicate that models face challenges in accurately retrieving and extracting data when it is nested across multiple levels.

## 5 Discussion

We aimed to investigate the capability of using LLM, RAG, and prompt engineering to rebuild the IDMP data model from free text documents in regulatory affairs. Our study confirmed that Claude Sonnet 3.5 can effectively extract IDMP-relevant data from unstructured SmPC files, especially with well-designed prompts like CARE and CIAEE. These prompts enhance the LLM's ability to extract exact tokens and capture semantic meaning from complex medical texts. BERT-based metrics showed a bimodal distribution of semantic similarity scores, clustering around values close to 1.0 (high similarity) and 0 (low similarity). This pattern resulted from prompt design and retrieval techniques. The RAG approach instructed the LLM to return an empty string if no relevant information was found, producing low similarity scores around 0. Rule-based retrieval ensured relevant information was present, but low similarity scores still occurred, indicating occasional extraction failures. Statistical analyses using the Kruskal-Wallis test confirmed that LLM, prompt design, embedding model, and retrieval technique significantly impacted performance metrics (*p* < 0.001). LLM and prompt designs had the highest *H* statistics, highlighting their substantial influence on structural and semantic similarity. This underscores the importance of selecting appropriate LLMs and designing effective prompts to optimize extraction performance.

A clear difference in performance between the LLM was evident: Claude 3.5 Sonnet generally produced higher similarity scores than Gemini 1.5 Flash, particularly in the rule-based retrieval context. This distinction was most pronounced in the rule-based retrieval results, where Gemini's similarity scores tended to cluster more frequently at the lower end compared to those of Claude. This suggests that while both models are capable, Claude 3.5 Sonnet has a superior ability to extract relevant information when provided with appropriate prompts and retrieval contexts. Our analysis indicated that the CARE prompt might consistently outperform other prompt designs, followed by CIAEE and then RISE. The superior performance of the CARE prompt underscores the potential significance of prompt engineering in optimizing LLM outputs. Specifically, CARE could achieve higher accuracy across key similarity metrics, suggesting that example-based prompts may effectively guide LLM to produce more precise and contextually appropriate responses. This is supported by radar chart comparisons, where CARE demonstrated the largest area, indicating better alignment with reference texts across various metrics. In contrast, the RISE prompt, despite its structured approach, might not perform as well due to the absence of example-based guidance, which appears crucial for optimal performance in this context.

These findings align with insights from related studies. Tang et al. (16) and Nori et al. (18) highlighted that incorporating examples and structured instructions can significantly enhance LLM performance in medical information extraction tasks. Zhou et al. (19) further validated that structured and adaptive prompts could benefit clinical relation extraction, supporting our approach. Additionally, Kartchner et al. (20) demonstrated the utility of context-rich and example-based prompts in zero-shot information extraction for clinical meta-analyses, reinforcing the efficacy of our prompting strategies. The implications of our findings suggest that carefully crafted, example-rich prompts like CARE might substantially improve LLM performance in complex, domain-specific tasks. This approach could reduce the reliance on extensive fine-tuning and expert-crafted prompts, potentially streamlining the deployment of LLM in real-world medical applications. Moreover, the lower performance of RISE indicates that while structured instructions are beneficial, they may not suffice without the inclusion of contextual examples.

The comparison between Semantic Search and Rule-Based methods revealed distinct strengths in different contexts. Rule-based approaches achieved higher precision in structured data extraction due to their reliance on predefined patterns. They consistently retrieved relevant context, leading to precise outputs by the LLM in predictable sections. However, they lacked adaptability when dealing with unstructured text.

Addressing the second research question, whether combining LLMs with rule-based methods can efficiently extract IDMP-relevant data, our findings confirm their complementary strengths. To assess the effectiveness of different retrieval techniques, we compared Semantic Search and Rule-Based approaches across key IDMP sections, as shown in the radar chart in Figure 8. Rule-Based retrieval demonstrated stronger performance in sections such as Therapeutic Indications, Ingredients Composition, and Packaged Medicinal Product, where information is typically localized within short, well-structured SmPC chapters. In contrast, Semantic Search outperformed in more semantically variable sections like Medicinal Product and Pharmaceutical Product, where relevant information is often implicit or scattered throughout longer textual content. Marketing Authorization Information showed comparable performance between both methods. This performance pattern is consistent with our mapping between IDMP fields and SmPC chapters. Many IDMP fields, such as Product Name, Pharmaceutical Form fields, Shelf Life, and Marketing Authorization Holder fields, are associated with short SmPC sections, where rule-based retrieval ensures high recall by directly mapping to the expected location. However, for fields like the ATC Code or those found in the Qualitative and Quantitative Composition chapter, which appear in longer and less structured sections, rule-based retrieval can reduce extraction quality. This is because entire chapters are passed to the LLM, potentially diluting the relevance of the target information and overwhelming the model's attention. Semantic Search, on the other hand, provides compact, contextually aligned chunks that maintain a balanced input size and higher information density.

Our evaluation of embedding models reveals that the generalist model e5-small-v2outperforms specialized models like ClinicalBERT, Bio_ClinicalBERT, and PharmBERT across most metrics. Its training on broader and more diverse datasets allows it to handle a wide range of language patterns and phrasing variations more effectively, making it more adaptable to the complexities found in SmPCs. This observation aligns with the findings of Excoffier et al. (29), who demonstrated that generalist embedding models surpass specialized clinical models in short-context clinical semantic search tasks due to their robustness against input variations.

However, in specific sections rich in medical terminology, such as “Therapeutic Indications,” specialized models performed comparably or even better than the generalist models. This suggests that while generalist models offer greater flexibility and extensive linguistic coverage, specialized models trained on medical terms enhance the ability to handle domain-specific language effectively. The specialized models' focused training on medical corpora enables them to capture subtle nuances and specific terminology that generalist models might overlook.

An in-depth analysis of exact matching in sections like “ATC Code” and “Marketing Authorization Number” further highlights the importance of embedding model selection based on content type. In the “ATC Code” section, the highest exact match similarity score was achieved by combining the Gemini 1.5 Flash LLM with the CARE prompt, using the Semantic Search retrieval technique and the S-PubMedBERT-MS-MARCO embedding model. This combination underscores the effectiveness of specialized embeddings in capturing the semantics of structured, domain-specific content where precise retrieval is critical. Conversely, in the “Marketing Authorization Number” section, the generalist model e5-small-v2 paired with the Claude 3.5 Sonnet LLM and the CARE prompt achieved high similarity scores. This indicates that generalist embeddings can be highly effective in contexts that require handling diverse language patterns and less specialized terminology. These findings emphasize that both generalist and specialized embedding models have their merits, and the optimal choice depends on the specific requirements of the content being processed. Generalist models like e5-small-v2 are advantageous for sections where linguistic diversity and adaptability are crucial. In contrast, specialized models excel in sections dense with medical terminology, benefiting from their focused training on domain-specific language. Our study underscores the importance of selecting appropriate embedding models based on the characteristics of the retrieval task. Aligning with prior research [see (23), (25), or (32)], embedding choice significantly affects retrieval outcomes, especially in specialized fields like healthcare and pharmaceuticals where precision is paramount. The integration of both generalist and specialized embeddings, tailored to specific sections of the SmPCs, can enhance the overall performance of retrieval systems, ensuring both adaptability and precision in handling complex medical documents.

Our analysis also showed that the CARE prompt consistently outperforms other prompt designs, followed by CIAEE and then RISE. The use of examples in CARE and CIAEE effectively guides the LLM to produce more accurate and contextually appropriate responses. The radar charts comparing prompt performance across key similarity metrics and IDMP sections highlighted that CARE's example-based structure provides clearer guidance, resulting in higher semantic similarity across metrics. Moreover, the section-wise analysis indicated that semantic similarity scores varied by section, with “Marketing Authorization Information” and “Therapeutic Indications” showing higher scores, while sections like “Pharmaceutical Product” and “Packaged Medicinal Product” had lower scores. This variation suggests that certain sections may inherently support more straightforward semantic alignment, possibly due to less complex language or more explicit structure.

We further analyzed the quality of data extraction based on the data level and degree of nesting, ranging from levels 1 to 4. The findings indicate a clear trend: as the level of nesting increases, the models' performance in accurately extracting and retrieving data decreases. This suggests that while LLM are adept at handling simple extraction tasks, they encounter significant challenges when dealing with deeply nested or complex hierarchical data. The implications of these results are critical for the application of LLM in domains requiring precise data structuring, such as the IDMP framework. The nested elements within the IDMP model necessitate not only the identification of individual data points but also the accurate reconstruction of hierarchical relationships between them. To mitigate these challenges, we employed prompt engineering techniques by incorporating examples in the CARE and CIAEE prompts to guide the LLM. Including examples was intended as part of the solution to enhance the models' ability to understand and extract nested information. Despite the additional support from the prompts, the models still struggled to interpret implicit information and establish dependencies among different elements. This indicates that while including examples in prompts is beneficial, it alone is not enough to address the inherent challenges posed by deeply nested data structures.

Looking ahead, a promising strategy would involve developing an adaptive orchestration framework that dynamically selects the optimal combination of LLM, prompt, retrieval technique, and embedding model tailored to the specific characteristics of each SmPC section. Our findings demonstrate that no single configuration performs best across all sections. While our current analysis focused on evaluating fixed combinations per experiment, we implicitly explored a hybrid strategy by analyzing performance variation across configurations and sections. However, a fully hybridized pipeline, capable of making real-time configuration decisions based on section metadata, content structure, or observed performance trends, was not implemented. In future work, we propose implementing a meta-controller or policy model trained on section-specific performance metrics to guide this selection process. Given the domain of regulatory affairs, where human validation is mandatory, this controller would operate within a human-in-the-loop framework. Extraction outputs would be systematically reviewed by regulatory experts, and their feedback used to iteratively refine the routing logic and improve extraction quality over time. An initial version of this framework could be prototyped using the gold standard dataset described in this study, serving as a foundation for testing different routing strategies. Over time, real-world usage data and expert feedback might allow for more sophisticated adaptation mechanisms. This conditional, feedback-driven approach would help balance the need for automation with the strict validation and accountability requirements of regulatory settings.

## 6 Conclusion

This study demonstrates the viability of integrating LLM, specifically Claude 3.5 Sonnet, with RAG to automate the extraction of IDMP-relevant data from unstructured SmPC documents. The findings underscore the importance of prompt engineering: example-rich prompts like CARE significantly enhance the LLM's ability to extract accurate and context-aware information. In comparative evaluations, Claude 3.5 Sonnet consistently outperformed Gemini 1.5 Flash, particularly when guided by well-crafted prompts and retrieval contexts. Our analysis also highlights the performance advantages of generalist embedding models in the semantic retrieval phase. In particular, e5-small-v2 delivered strong results across most metrics, demonstrating broad linguistic adaptability. However, specialized models such as ClinicalBERT and Bio_ClinicalBERT proved more effective in sections dense with medical terminology. These findings suggest that a hybrid strategy, combining generalist and domain-specific embeddings, can help tailor retrieval to the complexity of different content areas within SmPCs.

Future research should explore strategies for handling the challenges posed by complex SmPCs, especially those involving multiple dosage forms or intricate packaging configurations. Addressing the limitations of extracting deeply nested data structures may require hierarchical parsing techniques or the incorporation of richer contextual signals to support the LLM's reasoning. Evaluating open-source LLMs, enhancing RAG with re-ranking strategies, and experimenting with ensemble methods that combine multiple LLMs and embedding models could further improve accuracy and scalability. In addition, developing an adaptive pipeline that selects the optimal combination of models, prompts, and retrieval strategies for each section could significantly boost overall performance and reliability. Ultimately, the successful integration of Claude 3.5 Sonnet with RAG represents a significant advancement in automating the extraction of IDMP-relevant data from unstructured regulatory documents. By enabling accurate, context-aware, and scalable data extraction from SmPCs, this approach contributes meaningfully to the informatization of pharmaceutical regulatory affairs. It not only streamlines data standardization and harmonization but also supports regulatory compliance and interoperability across global health authorities.

## Data Availability

The datasets presented in this article are not readily available because the generated datasets are proprietary to the company and are integral to confidential customer business processes. As such, they cannot be shared outside the organization due to contractual and confidentiality obligations.

## Footnotes

^1^ https://gitlab.com/hockadi/idmp-extraction

^2^Available at: https://aws.amazon.com/about-aws/whats-new/2024/03/anthropics-claude-3-sonnet-model-amazon-bedrock/.

^3^https://python.langchain.com/docs/integrations/vectorstores/chroma/. ChromaDB is an open-source embedding database integrated with LangChain for storing and retrieving vectorized document chunks.

## 7 Annex

### 7.1 Annex 1. Fields constituting the minimal IDMP

Section 1.—Medical Product—contains foundational fields, namely:

- **Product Name**: identifying the medicinal product.
- **ATC Code**: Provides the Anatomical Therapeutic Chemical classification system name, ensuring clear identification of the drugs therapeutic group and its usage.
- **Authorized Pharmaceutical Form**: the marketed form of the medicinal product.

Section 2.—Marketing authorization information— captures all the medicinal product information required to comply with the regulatory requirements for market entry and maintenance. This section focuses on regulatory fields such as:

- **Country or Regulatory Region**: specifies if it is a Centralized Authorized Product (EU-level) or National Authorized Product (National level) medicinal product;
- **Marketing Authorization Number**: a unique identifier for regulatory approval at the product or package level;
- **Date of First Authorization**: represents the regulatory milestone marking the product's initial approval for market entry;
- **Marketing Authorization Holder (MAH)**: the entity responsible for the medicinal product, including

- **MAH Address**: street name, postal code, city, and country;
- **MAH Name**.

Section 3. Therapeutic indications it cover the known therapeutic indications for which the medicinal product is authorized.

Section 4. The packaged medicinal product section—is inherently complex due to its multiple nested levels, reflecting the hierarchy of packaging information from the overall package description to the item components and materials used. It includes:

- **Package Description**: details of the overall physical packaging;
- **Package Composition**:

**Container Type**: specifies the type of packaging.
**Container Description**: specifies the container description.
**Package Item Material**: material of the overall physical packaging.
**Package Component**:

**Material**: the specific material used for the container type.
**Value**: details of quantity or material attributes.

**Shelf life**:

**Value**: specifies the shelf-life value (in years, months, hours) with respect to storage type.
**Special precautions for storage**: specifies the special conditions for storage of the medicinal product.

Section 5.—The ingredients—details the chemical composition of the medicinal product, including active ingredients and excipients:

- **Composition Active**:

**Active Substance Salt**: specifies the salt composition of the active ingredient, with a value and its dosage.
• **Active Substance Base**: the main component of the medicinal product, distinguished from the salt form, with a value and its dosage.

**Composition excipient**: lists excipients and their dosage.

Section 6.—The pharmaceutical product—ensures that all product configurations are well documented to align with regulatory and clinical requirements. For instance, the dose form can affect the efficacy of the medicinal product. The fields include:

- **Administrable Dose Form**: specifies the form in which the product can be administered.
- **Route of Administration**: specifies how the product should be administered.
- **Unit of Presentation**: specifies how the medicinal product is presented in a measurable form.
